# Supplementary material for: Regional Molecular Mapping of Primate Synapses during Normal Healthy Aging
Source: Cell Rep. 2019 Apr 23;27(4):1018–1026.e4. doi: 10.1016/j.celrep.2019.03.096 (PMC6486486; doi:10.1016/j.celrep.2019.03.096)
Supplement: Document S1. Figures S1–S4 [file mmc1.pdf]

Cell Reports, Volume *27*

## Supplemental Information

# Regional Molecular Mapping of Primate Synapses during Normal Healthy Aging

**Laura C. Graham, Michael J. Naldrett, Steven G. Kohama, Colin Smith, Douglas J. Lamont, Barry W. McColl, Thomas H. Gillingwater, Paul Skehel, Henryk F. Urbanski, and Thomas M. Wishart**

**A.** NHP ageing timecourse enrichment analysis

| PANTHER GO-Slim Cellular Component | Fold Enrichment | P-value |
|---|---|---|
| Synapse | 5.72 | 4.05E-03 |
| Vesicle coat | 4.57 | 2.3E-04 |
| Mitochondrial inner membrane | 3.63 | 6.73E-09 |
| Presynaptic membrane | 3.48 | 2.21E-02 |
| Heterotrimeric G-protein complex | 3.43 | 1.33E-02 |
| SNARE complex | 3.3 | 9.26E-03 |
| Postsynaptic membrane | 3.2 | 1.06E-02 |
| Cytoplasmic membrane-bound vesicle | 3.05 | 4.16E-08 |
| Endosome | 2.91 | 3.2E-05 |

**B.** Human patient ageing timecourse enrichment analysis

| PANTHER GO-Slim Cellular Component | Fold enrichment | P-value |
|---|---|---|
| Postsynaptic membrane | 10.48 | 3.97E-02 |
| Mitochondrial inner membrane | 5.69 | 4.06E-09 |
| Ribosome | 3.81 | 2.7E-10 |
| Actin cytoskeleton | 3.57 | 1.02E-12 |
| Cytosol | 3.4 | 2.07E-21 |
| Cell junction | 2.89 | 8.1E-03 |
| Microtubule | 2.55 | 5.63E-03 |
| Cytoskeleton | 1.96 | 1.53E-05 |
| Cytoplasm | 1.93 | 3.66E-29 |

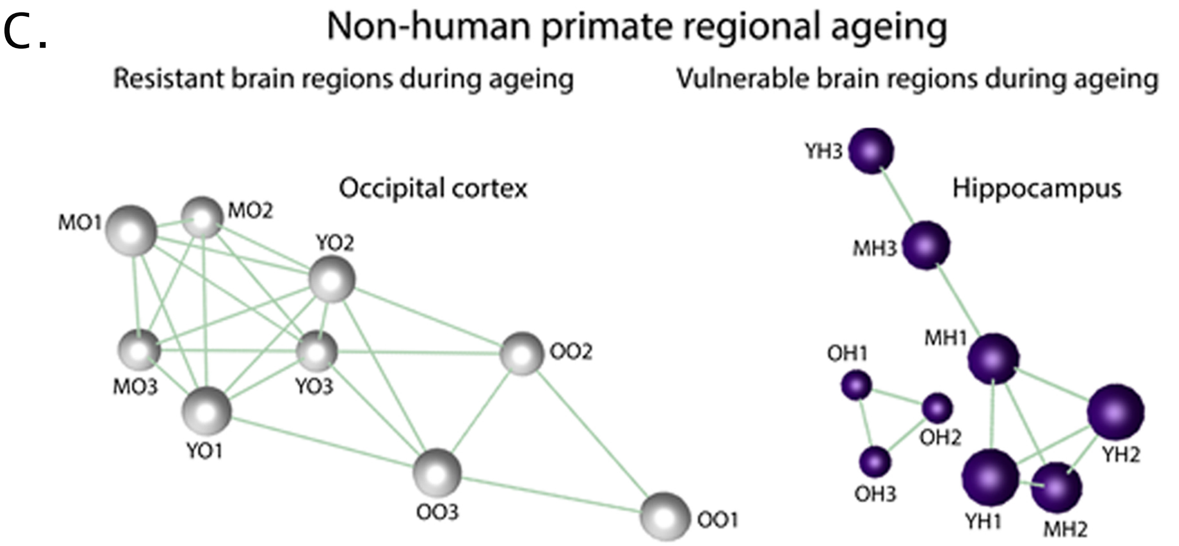


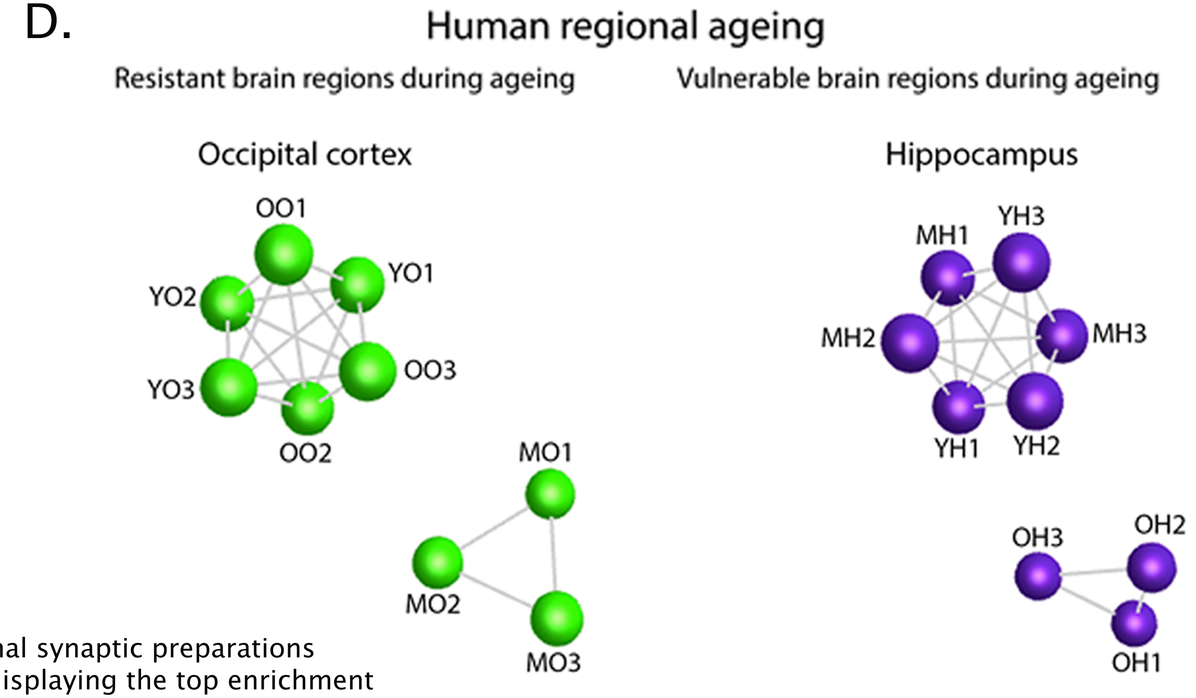


Figure S1 Enrichment analyses demonstrating purity of NHP and human regional synaptic preparations (A&B related to Figure 1): PANTHER Gene ontology (GO) enrichment analyses displaying the top enrichment terms in both the young NHP synaptosomal preparations (A.) and the young human patient synaptic isolates (B.). Synaptic ageing is regionally heterogeneous. (C&D related to Figure 2): Unbiased sample-sample Pearson correlation analyses generated from BioLayout Express3D software. C. NHP hippocampal and occipital cortex synaptic timecourse Pearson correlation analyses. DA.. NHP hippocampal and occipital cortex synaptic timecourse Pearson correlation analyses. Nodes signify individual samples and edges reflect the strength of correlation of expression. Increased edge length between nodes represents reduced correlative value between sample nodes; reduced edge length between nodes represents increased correlative value between sample nodes; detachment of nodes from primary cluster illustrates significant variability between sample populations. Graphs clustered using Pearson r=0.98.

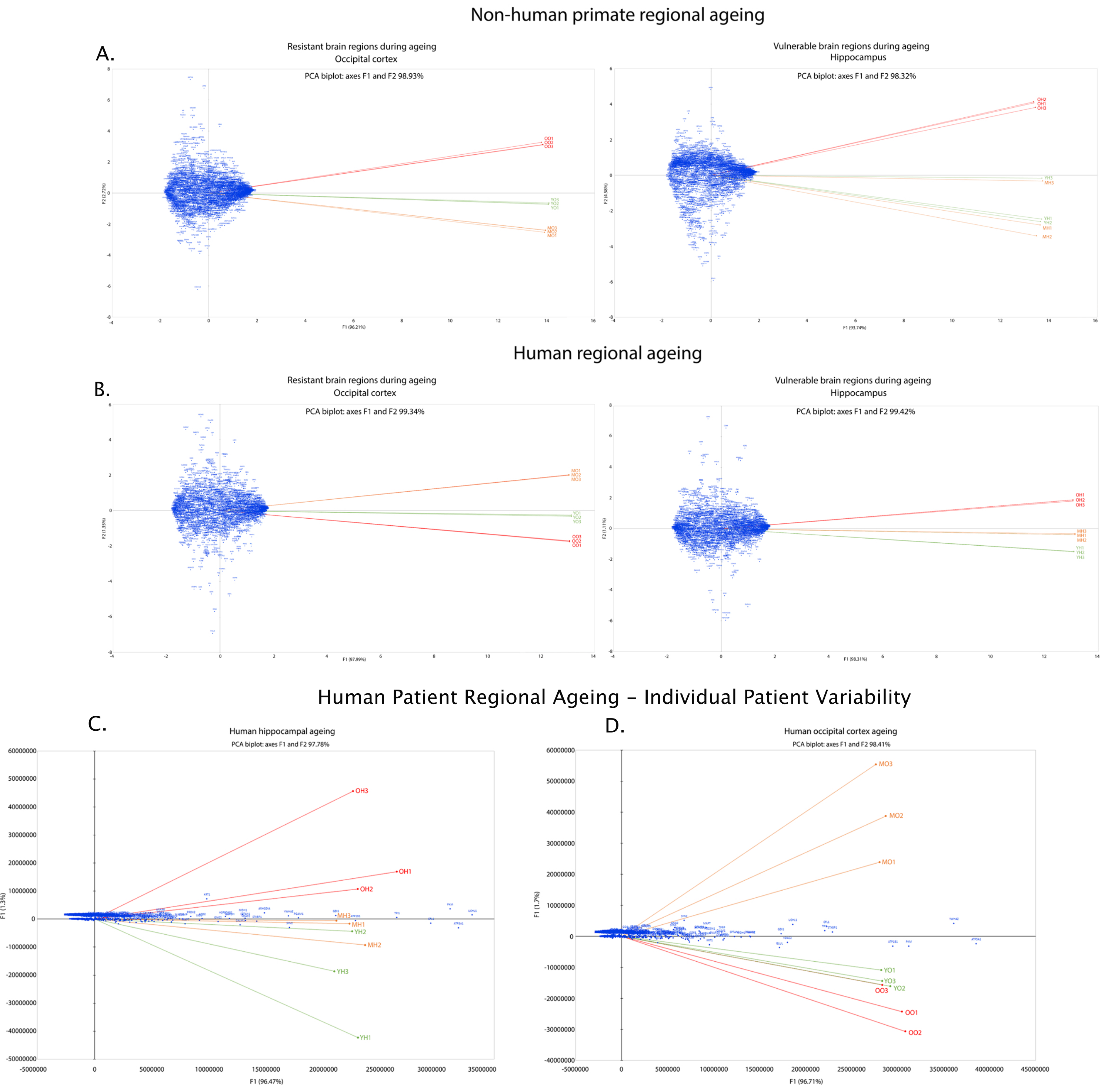


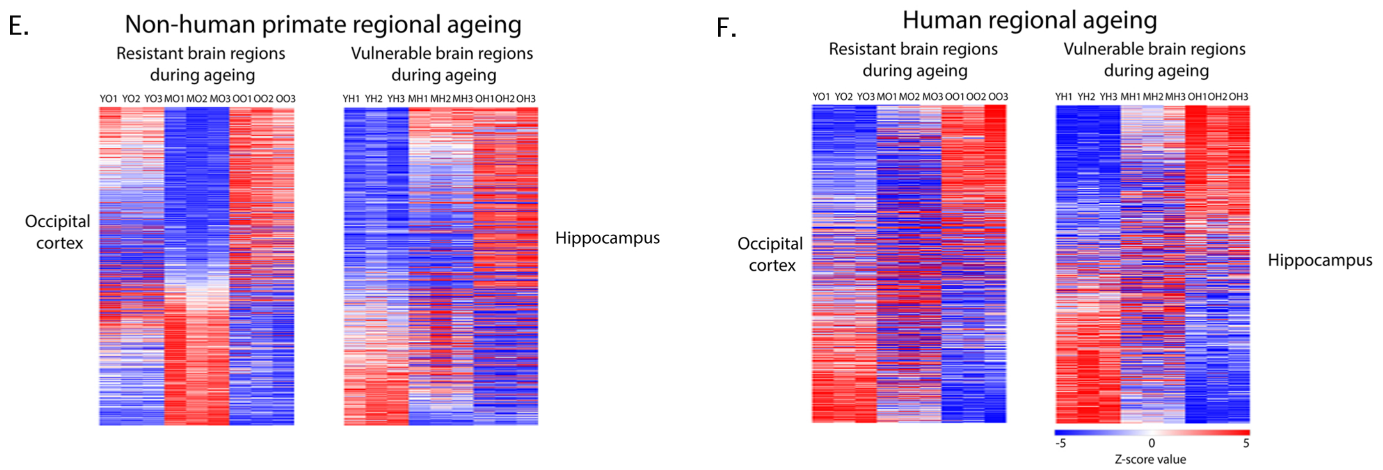


Figure S2: PCA biplots of sample variability (technical replicates – A&B; Example biological replicates – C&D) and heat maps of temporal expression (E&F): PCA biplots conveying global variability in the NHP and human patient brain regional proteomic datasets (related to Figure 2): PCA biplots display identified proteins from the NHP (A) and human patient (B) occipital cortex and hippocampal proteomic datasets. Individual sample technical replicates are indicated by filled circles and grouped by colour. Patient sample technical replicates are indicated by filled circles and grouped by colour: green = young patients; orange = mid-age patients; red = aged patients. Sample variability is dictated by the location of the representative markers in space: proximate variables demonstrate a significant positive correlation (r value is close to or equal to 1); variables opposing the centre line display a significant negative correlation (r value is close to or equal to -1). YO = young occipital cortex; MO = mid-age occipital cortex; OO = old occipital cortex; YH = young hippocampus; MH = mid-age hippocampus; OH = old hippocampus; numbers refer to technical replicates. Blue = active observations in the PCA space (proteins identified by gene name); green, orange and red = active variables observed in the PCA space (green = young time point; orange = mid-age time point; red = aged time point). All PCA biplots generated using Pearson correlation.
C&D (related to Figure 2): PCA biplots displaying inter-patient variability in the human ageing regional proteomic datasets. PCA biplots display 630 identified proteins from the human patient hippocampal (C.) and occipital cortex (D.) proteomic datasets. See above for colour code, abbreviations and generation. Numbers refer to biological replicates. E&F (related to Figure 2): Heat maps illustrating temporal protein expression in differentially vulnerable populations of synapses derived from the NHP and human patient brain. E. Heat maps displaying temporal protein expression in NHP occipital cortex (left panel) and hippocampal (right panel) synaptic populations across ageing.
F. Temporal protein expression in human patient occipital cortex (left panel) and hippocampal (right panel) synapses during advancing age. Heat maps display Z-score transformed normalized abundance values derived from the raw proteomic data. Blue = minimum Z-score value (-5); red = maximum Z-score value (5). YO = young occipital cortex; MO = mid-age occipital cortex; OO = old occipital cortex; YH = young hippocampus; MH = mid-age hippocampus; OH = old hippocampus; numbers refer to technical replicates.

## A. Correlates of ageing

### NHP Hippocampus

**Steady increase during ageing**

| PANTHER GO Molecular Function | Fold Enrichment | P-value |
|---|---|---|
| Phosphatidylcholine-sterol activity | > 100 | 1.07E-05 |
| Low-density lipoprotein particle binding | 33.53 | 1.64E-04 |
| Amyloid-beta binding | 22.01 | 5.83E-06 |
| Cholesterol binding | 20.76 | 7.56E-06 |
| Calcium-dependent protein binding | 19.63 | 9.67E-06 |
| Oxidoreductase activity | 11.18 | 1.03E-06 |
| Cadherin binding | 6.72 | 3.55E-05 |
| Cell adhesion molecule binding | 6.05 | 3.11E-06 |
| Actin binding | 5.04 | 1.64E-05 |

**Steady decrease during ageing**

| PANTHER GO Molecular Function | Fold Enrichment | P-value |
|---|---|---|
| Malate dehydrogenase activity | > 100 | 1.49E-04 |
| Oxidoreductase activity, acting on NAD(P)H | 17.86 | 7.01E-04 |
| NADH dehydrogenase activity | 34.44 | 1.76E-03 |
| Oxidoreductase activity | 3.99 | 3.76E-03 |
| Ferrochelatase activity | > 100 | 4.14E-03 |
| Citrate synthase activity | > 100 | 4.14E-03 |
| Lactoylglutathione lyase activity | > 100 | 4.14E-03 |
| Methionine-tRNA ligase activity | > 100 | 6.20E-03 |
| 3-oxoacid CoA-transferase activity | > 100 | 6.20E-03 |

### NHP Occipital cortex

**Steady increase during ageing**

| PANTHER GO Molecular Function | Fold Enrichment | P-value |
|---|---|---|
| Very-low-density lipoprotein particle binding | > 100 | 3.28E-05 |
| Phosphatidylcholine-sterol activity | > 100 | 3.28E-05 |
| Amide binding | 15.07 | 1.49E-04 |
| Antioxidant activity | 30.13 | 1.52E-04 |
| Low-density lipoprotein particle binding | > 100 | 2.28E-04 |
| Cholesterol transporter activity | 63.13 | 5.45E-04 |
| Sterol transporter activity | 57.64 | 6.45E-04 |
| Sulfur compound binding | 18.08 | 6.50E-04 |
| Oxidoreductase activity | 5.49 | 6.85E-04 |

**Steady decrease during ageing**

| PANTHER GO Molecular Function | Fold Enrichment | P-value |
|---|---|---|
| Electron transporter | > 100 | 1.08E-05 |
| Proton transmembrane transporter activity | 20.90 | 4.22E-05 |
| Transmembrane transporter activity | 5.54 | 2.05E-04 |
| Nucleoside monophosphate kinase activity | 73.14 | 4.09E-04 |
| Inorganic molecule transporter activity | 6.00 | 4.13E-04 |
| Transporter activity | 4.82 | 4.74E-04 |
| Phosphotransferase activity | 43.03 | 1.10E-03 |
| Monocarboxylic acid transporter activity | 40.64 | 1.23E-03 |
| Cation transmembrane transporter activity | 8.48 | 1.23E-03 |

### Human Hippocampus

**Steady increase during ageing**

| PANTHER GO Molecular Function | Fold Enrichment | P-value |
|---|---|---|
| Myosin II binding | 54.80 | 4.88E-05 |
| Aldehyde dehydrogenase (NAD) activity | 29.23 | 2.33E-04 |
| Fatty acid binding | 16.70 | 1.40E-04 |
| Calcium-dependent protein binding | 14.14 | 6.87E-06 |
| Amyloid-beta binding | 12.82 | 6.41E-05 |
| NAD binding | 12.38 | 7.47E-05 |
| Oxidoreductase activity | 10.74 | 6.19E-08 |
| Magnesium ion binding | 6.99 | 2.61E-06 |
| Cadherin binding | 4.65 | 7.67E-05 |

**Steady decrease during ageing**

| PANTHER GO Molecular Function | Fold Enrichment | P-value |
|---|---|---|
| NADH dehydrogenase (quinone) activity | 49.72 | 4.46E-14 |
| Oxidoreductase activity | 38.12 | 4.73E-13 |
| Catalytic activity | 1.95 | 8.07E-08 |
| Electron transfer activity | 14.17 | 9.38E-07 |
| Cytochrome-c oxidase activity | 33.88 | 9.67E-06 |
| Heme-copper terminal oxidase activity | 33.88 | 9.67E-06 |
| Proton transmembrane transporter activity | 10.02 | 3.82E-05 |
| Metal cluster binding | 13.86 | 2.48E-04 |
| Structural constituent of presynapse | > 100 | 2.78E-04 |

### Human Occipital cortex

**Steady increase during ageing**

| PANTHER GO Molecular Function | Fold Enrichment | P-value |
|---|---|---|
| Antioxidant activity | 19.04 | 8.51E-06 |
| Oxidoreductase activity | 4.70 | 2.08E-05 |
| Identical protein binding | 2.74 | 2.77E-04 |
| Coenzyme binding | 6.65 | 3.09E-04 |
| Chaperone binding | 12.82 | 3.10E-04 |
| NAD binding | 16.46 | 9.26E-04 |
| Copper ion binding | 15.66 | 1.06E-03 |
| Cofactor binding | 4.37 | 1.15E-03 |
| Catalytic activity | 1.67 | 1.32E-03 |

**Steady decrease during ageing**

| PANTHER GO Molecular Function | Fold Enrichment | P-value |
|---|---|---|
| Oxidoreductase activity | 7.33 | 8.53E-12 |
| NADH dehydrogenase activity | 43.53 | 6.20E-10 |
| Catalytic activity | 1.95 | 2.33E-06 |
| Electron transfer activity | 15.52 | 3.26E-06 |
| Cofactor binding | 5.63 | 1.19E-05 |
| Glutamate:sodium symporter activity | > 100 | 2.38E-04 |
| Pyruvate dehydrogenase activity | 97.42 | 3.17E-04 |
| Coenzyme binding | 6.01 | 5.38E-04 |
| Glutamate receptor binding | 18.27 | 7.04E-04 |

## B. Correlates of vulnerability

### NHP Hippocampus

**Late stage increase during ageing**

| PANTHER GO Molecular Function | Fold Enrichment | P-value |
|---|---|---|
| Cadherin binding | 21.51 | 5.29E-11 |
| Cell adhesion molecule binding | 14.10 | 2.71E-09 |
| Phospholipase A2 inhibitor activity | > 100 | 4.22E-05 |
| S100 protein binding | > 100 | 1.05E-04 |
| Calcium ion binding | 5.31 | 1.30E-04 |
| Calcium-dependent phospholipid binding | 31.90 | 1.44E-04 |
| Phospholipase inhibitor activity | > 100 | 1.47E-04 |
| Water channel activity | > 100 | 2.51E-04 |
| Water transmembrane transporter activity | 93.04 | 3.13E-04 |

**Late stage decrease during ageing**

| PANTHER GO Molecular Function | Fold Enrichment | P-value |
|---|---|---|
| Dynein intermediate chain binding | 41.92 | 1.23E-03 |
| Protein binding | 1.93 | 3.23E-03 |
| Guanine deaminase activity | > 100 | 4.32E-03 |
| Prenylcysteine oxidase activity | > 100 | 6.48E-03 |
| Calcium ion binding | 4.11 | 7.33E-03 |
| Glutaminase activity | > 100 | 8.63E-03 |
| Ganglioside GT1b binding | > 100 | 8.63E-03 |
| Sialic acid binding | > 100 | 8.63E-03 |
| Oxidoreductase activity | > 100 | 8.63E-03 |

### NHP Occipital cortex

**Late stage increase during ageing**

| PANTHER GO Molecular Function | Fold Enrichment | P-value |
|---|---|---|
| Catalytic activity | 1.75 | 9.21E-05 |
| Oxidoreductase activity | 11.21 | 1.05E-04 |
| Transferase activity | > 100 | 1.08E-04 |
| ATPase regulator activity | 34.96 | 1.26E-04 |
| Cytoskeletal protein binding | 3.63 | 4.71E-04 |
| Ribose phosphate diphosphokinase activity | 58.27 | 7.94E-04 |
| Protein binding | 1.69 | 1.23E-03 |
| Peptidase activity | 3.44 | 1.30E-03 |
| ATPase activator activity | 38.85 | 1.59E-03 |

**Late stage decrease during ageing**

| PANTHER GO Molecular Function | Fold Enrichment | P-value |
|---|---|---|
| Calmodulin-dependent protein kinase activity | 42.54 | 6.83E-05 |
| Rac GTPase binding | 25.29 | 2.83E-04 |
| Clathrin binding | 20.80 | 4.87E-04 |
| Rho GTPase binding | 9.82 | 8.24E-04 |
| Cytoskeletal protein binding | 3.88 | 1.08E-03 |
| Spectrin binding | 36.70 | 1.66E-03 |
| ATP-dependent microtubule motor activity | 28.36 | 2.65E-03 |
| Dynein intermediate chain binding | 28.36 | 2.65E-03 |
| Glutamate receptor activity | 24.00 | 3.60E-03 |

### Human Hippocampus

**Late stage increase during ageing**

| PANTHER GO Molecular Function | Fold Enrichment | P-value |
|---|---|---|
| Phospholipase inhibitor activity | 43.12 | 8.08E-05 |
| S100 protein binding | 34.50 | 1.43E-04 |
| Lipase inhibitor activity | 30.44 | 1.98E-04 |
| Calcium-dependent protein binding | 13.91 | 4.25E-05 |
| Cadherin binding | 8.79 | 7.86E-11 |
| Actin binding | 7.27 | 9.23E-11 |
| Cell adhesion molecule binding | 7.16 | 9.84E-12 |
| Calcium ion binding | 4.39 | 1.83E-07 |
| Cytoskeletal protein binding | 4.05 | 2.71E-08 |

**Late stage decrease during ageing**

| PANTHER GO Molecular Function | Fold Enrichment | P-value |
|---|---|---|
| Protein binding | 1.54 | 2.10E-12 |
| Cytoskeletal protein binding | 3.90 | 1.09E-07 |
| ATP-dependent protein binding | 65.21 | 2.91E-05 |
| Structural constituent of ribosome | 7.25 | 6.71E-05 |
| Tubulin binding | 4.74 | 1.46E-04 |
| Glutamate receptor binding | 14.49 | 2.23E-04 |
| Glutaminase activity | > 100 | 3.21E-04 |
| Chaperone binding | 8.61 | 3.60E-04 |
| Protein self-association | 11.79 | 4.67E-04 |

### Human Occipital cortex

**Late stage increase during ageing**

| PANTHER GO Molecular Function | Fold Enrichment | P-value |
|---|---|---|
| Structural constituent of ribosome | 9.33 | 9.21E-10 |
| RNA binding | 2.77 | 2.80E-09 |
| Identical protein binding | 2.59 | 1.77E-08 |
| Catalytic activity | 1.68 | 2.31E-08 |
| Cadherin binding | 5.35 | 2.59E-07 |
| Structural molecule activity | 3.30 | 4.31E-07 |
| Protein homodimerization activity | 3.16 | 8.74E-07 |
| Oxidoreductase activity | 8.19 | 2.75E-06 |
| Ion binding | 1.49 | 2.81E-05 |

**Late stage decrease during ageing**

| PANTHER GO Molecular Function | Fold Enrichment | P-value |
|---|---|---|
| Cytoskeletal protein binding | 5.37 | 9.97E-11 |
| Structural constituent of synapse | 57.18 | 1.54E-06 |
| Protein binding | 1.42 | 4.01E-06 |
| Catalytic activity | 1.76 | 1.41E-05 |
| Actin filament binding | 8.43 | 2.47E-05 |
| Calmodulin binding | 8.38 | 2.55E-05 |
| Proline-rich region binding | 34.31 | 1.33E-04 |
| Clathrin binding | 15.51 | 1.65E-04 |
| Structural molecule activity | 3.37 | 2.44E-04 |

Figure S3 (related to Figure 3): Enrichment analyses displaying the predominant molecular functions of identified Biolayout Express3D clusters. PANTHER GO Enrichment tables demonstrating the primary molecular functions of identified clusters termed “biomarkers of ageing” (A.) or “biomarkers of vulnerability” (B.) in synaptic isolates derived from both the NHP and human patient hippocampus and occipital cortex. Note the difference in enrichment terms between resistant (occipital cortex) and vulnerable (hippocampus) brain regions within species. Additionally, note the identification of similar enrichment terms in analogous brain regions independent of species.

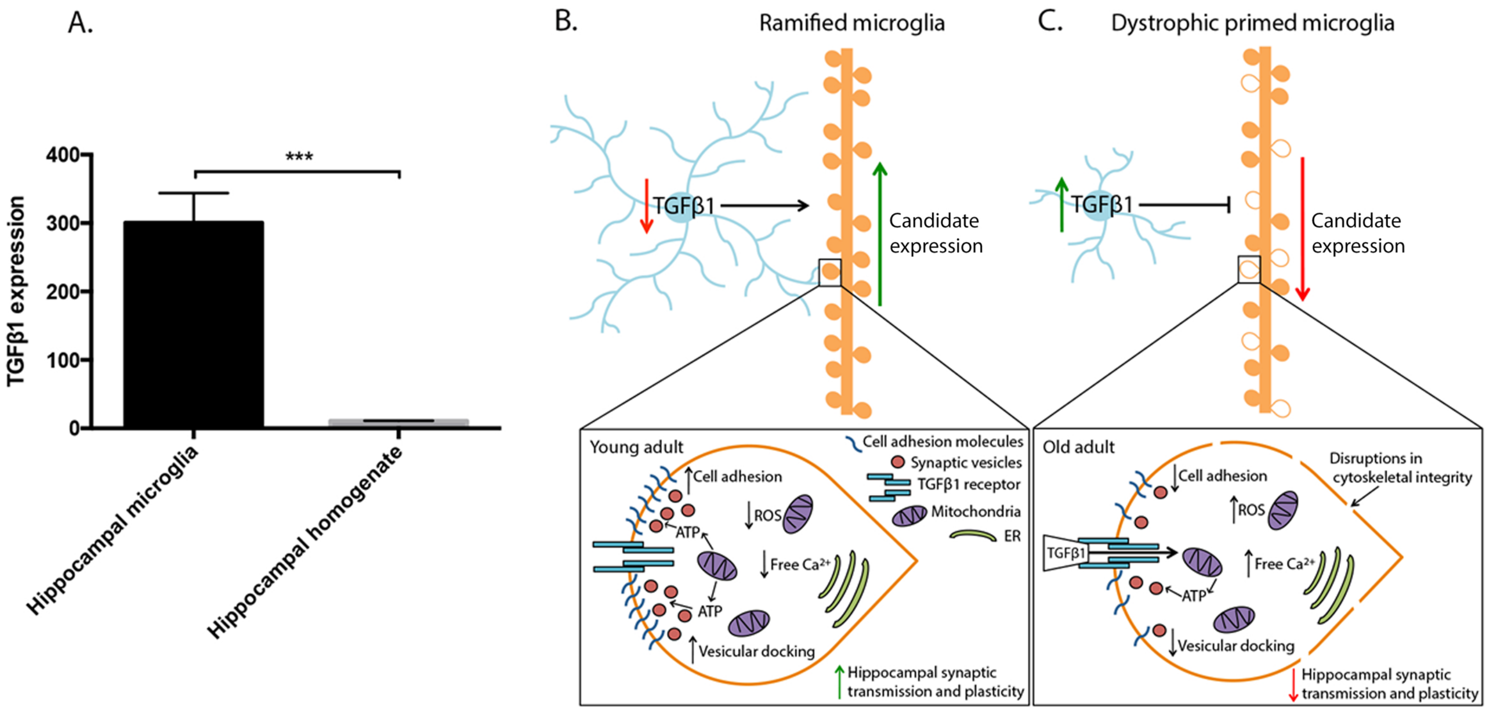


Figure S4 (related to Figure 4): TGFβ1 is expressed exclusively in microglial isolates. A. Bar chart displaying TGFβ1 transcript expression in isolated hippocampal microglia and hippocampal homogenate. The homogenate exhibits relatively little TGFβ1 expression versus the isolated microglia (unpaired two-tailed student's t-tests, $p=0.0009$; data represent mean  SEM). B&C. Summary of synaptic-microglial interactions and regulation of the cellular milieu during ageing. Young adult microglia (B.) demonstrate ramified morphology and inhibition of the TGFβ1 signalling cascade, promoting homeostatic regulation of the subcellular machinery within the synaptic compartment. Intracellular processes are illustrated in the black box. Conversely, resident microglia in an aged brain display a dystrophic, primed phenotype (C.). Activation of the TGFβ1 signalling cascade, by binding of TGFβ1 to the TGFβ1 synaptic receptor, promotes injurious events within the synaptic terminal facilitating synapse instability. Orange colour represents dendritic arbour with synaptic terminals; pale blue indicates microglia; white synaptic terminals symbolises dysfunction. See key in black box for intracellular information. YH – young hippocampus; MH – mid-age hippocampus; OH – old hippocampus; YO – young occipital; MO – mid-age occipital; OO – old age occipital.
