# Supplementary material for: Regional Molecular Mapping of Primate Synapses during Normal Healthy Aging
Source: Cell Rep. 2019 Apr 23;27(4):1018–1026.e4. doi: 10.1016/j.celrep.2019.03.096 (PMC6486486; doi:10.1016/j.celrep.2019.03.096)
Supplement: Document S2. Article plus Supplemental Information [file mmc2.pdf]

Cell Reports

Report

Regional Molecular Mapping of Primate Synapses during Normal Healthy Aging

Graphical Abstract

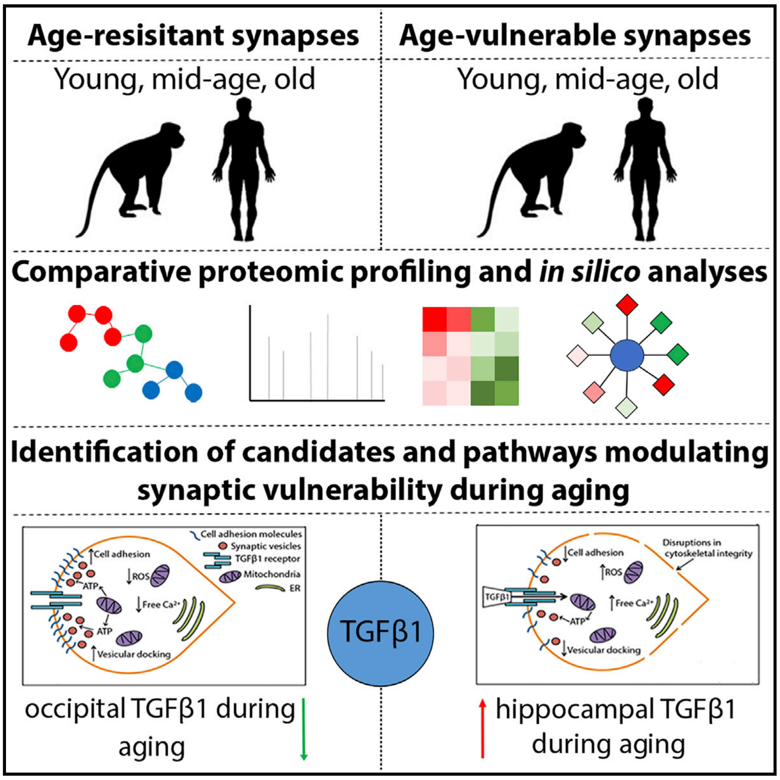

Highlights

- Discrete synaptic populations age in a region-dependent manner
- Regional heterogeneity in the synaptic proteome is conserved between humans and NHPs
- TGF-β1 signaling correlates with regional synaptic vulnerability in aged primate species

Authors

Laura C. Graham, Michael J. Naldrett, Steven G. Kohama, ..., Paul Skehel, Henryk F. Urbanski, Thomas M. Wishart

Correspondence

t.m.wishart@roslin.ed.ac.uk

In Brief

Graham et al. reveal the regional heterogeneity of synaptic aging and how proteostatic alterations may dictate selective synaptic vulnerability. The authors use comparative proteomic profiling to identify microglial TGF-β1 signaling as a conserved upstream modulator of regional synaptic cascades during aging in both humans and non-human primates (NHPs).

Graham et al., 2019, Cell Reports *27*, 1018–1026
April 23, 2019 © 2019 The Author(s).
https://doi.org/10.1016/j.celrep.2019.03.096

CellPress

OPEN ACCESS
CellPress

Cell Reports
Report

Regional Molecular Mapping of Primate Synapses during Normal Healthy Aging

Laura C. Graham,[1,2] Michael J. Naldrett,[3,4] Steven G. Kohama,[5] Colin Smith,[2,6] Douglas J. Lamont,[7] Barry W. McColl,[1,8] Thomas H. Gillingwater,[2,6] Paul Skehel,[2,9] Henryk F. Urbanski,[5] and Thomas M. Wishart[1,2,8,10,*]

[1]The Roslin Institute, University of Edinburgh, Easter Bush Campus, Midlothian, Edinburgh EH25 9RG, UK
[2]Euan MacDonald Centre, Chancellor's Building, University of Edinburgh, 49 Little France Crescent, Edinburgh EH16 4SB, UK
[3]Donald Danforth Plant Science Center, 975 N Warson Rd., St. Louis, MO 63132, USA
[4]Proteomics and Metabolomics Facility, Center for Biotechnology, Beadle Center, University of Nebraska, Lincoln, 1901 Vine St., Lincoln, NE 68588, USA
[5]Oregon National Primate Research Center, 505 NW 185th Ave., Beaverton, OR 97006, USA
[6]Edinburgh Medical School, Biomedical Sciences, Hugh Robson Building, George Square, Edinburgh EH8 9XD, UK
[7]FingerPrints Proteomic Facility, College of Life Sciences, University of Dundee, Dow Street, Dundee DD1 5EH, UK
[8]Centre for Dementia Prevention, University of Edinburgh, 9A Bioquarter, 9 Little France Road, Edinburgh EH16 4UX, UK
[9]Centre for Discovery Brain Sciences, University of Edinburgh, Hugh Robson Building, Edinburgh EH8 9XD, UK
[10]Lead Contact
*Correspondence: t.m.wishart@roslin.ed.ac.uk
https://doi.org/10.1016/j.celrep.2019.03.096

SUMMARY

Normal mammalian brain aging is characterized by the selective loss of discrete populations of dendritic spines and synapses, particularly affecting neuroanatomical regions such as the hippocampus. Although previous investigations have quantified this morphologically, the molecular pathways orchestrating preferential synaptic vulnerability remain to be elucidated. Using quantitative proteomics and healthy rhesus macaque and human patient brain regional tissues, we have comprehensively profiled the temporal expression of the synaptic proteome throughout the adult lifespan in differentially vulnerable brain regions. Comparative profiling of hippocampal (age vulnerable) and occipital cortex (age resistant) synapses revealed discrete and dynamic alterations in the synaptic proteome, which appear unequivocally conserved between species. The generation of these unique and important datasets will aid in delineating the molecular mechanisms underpinning primate brain aging, in addition to deciphering the regulatory biochemical cascades governing neurodegenerative disease pathogenesis.

INTRODUCTION

The loss and dysfunction of selected populations of synapses is a characteristic feature of mammalian brain aging, and alterations in these receptive compartments are considered to underpin age-related cognitive decline (Morrison and Baxter, 2012; Burke and Barnes, 2006; Small et al., 2004; Peters, 2002; Hof et al., 2000, 2002). It is well established that the hippocampal synaptic architecture displays a particular vulnerability to a range of stimuli during advancing age (Morrison and Baxter, 2012; Burke and Barnes, 2006; Small et al., 2004), whereas populations of synapses resident to the occipital cortex exhibit resistance to age-related alterations (Peters, 2002; Hof et al., 2000, 2002; Papazafiri et al., 1995; Braak and Braak, 1995; Clare et al., 2010; Clinton et al., 1994; Pikkarainen et al., 2009; Josephs, 2007; Fjell et al., 2015). Although the synaptic alterations underpinning age-related cognitive decline differ from the extensive neuronal loss that leads to dementia and Alzheimer's disease (AD), these alterations may render neurons more vulnerable to degeneration during the aging process (Morrison and Baxter, 2012; Gillingwater and Wishart, 2013). An essential area for investigation is to determine how synaptic alterations may leave a neuron vulnerable to neurodegeneration and the pathological substrates promoting such vulnerability. A great deal of our knowledge concerning the mechanisms of cognitive and brain aging has been provided by rodent studies. Several investigations have reported heterogeneous expression of the hippocampal synaptic proteome in aging rodents (Végh et al., 2014; Sato et al., 2005; VanGuilder et al., 2010); however, it has been established that relatively few age-related gene and protein expression alterations demonstrate conservation from mouse to man (Loerch et al., 2008). Evolutionary divergence of the primate cortex has promoted functional neuronal alterations that are uniquely primate (Morrison and Baxter, 2012), and studies documenting rodent biological brain aging are not necessarily representative of the complex processes occurring in the human patient, particularly with regards to selective synaptic vulnerability (Loerch et al., 2008). Thus, to tease apart human age-related cognitive decline, non-human primates (NHPs) provide obvious advantages. NHPs are phylogenetically closer to humans and possess distinctly primate morphological, endocrine, behavioral, and cognitive traits (Morrison and Baxter, 2012; Hof et al., 2002), in addition to an increased lifespan, which may provide data uniquely relevant to human aging.

In what appears to be the first investigation into the regional diversity of the primate synaptic proteome during the adult lifespan, we present a comprehensive synaptic atlas supporting

 © 2019 The Author(s).
This is an open access article under the CC BY license (http://creativecommons.org/licenses/by/4.0/).

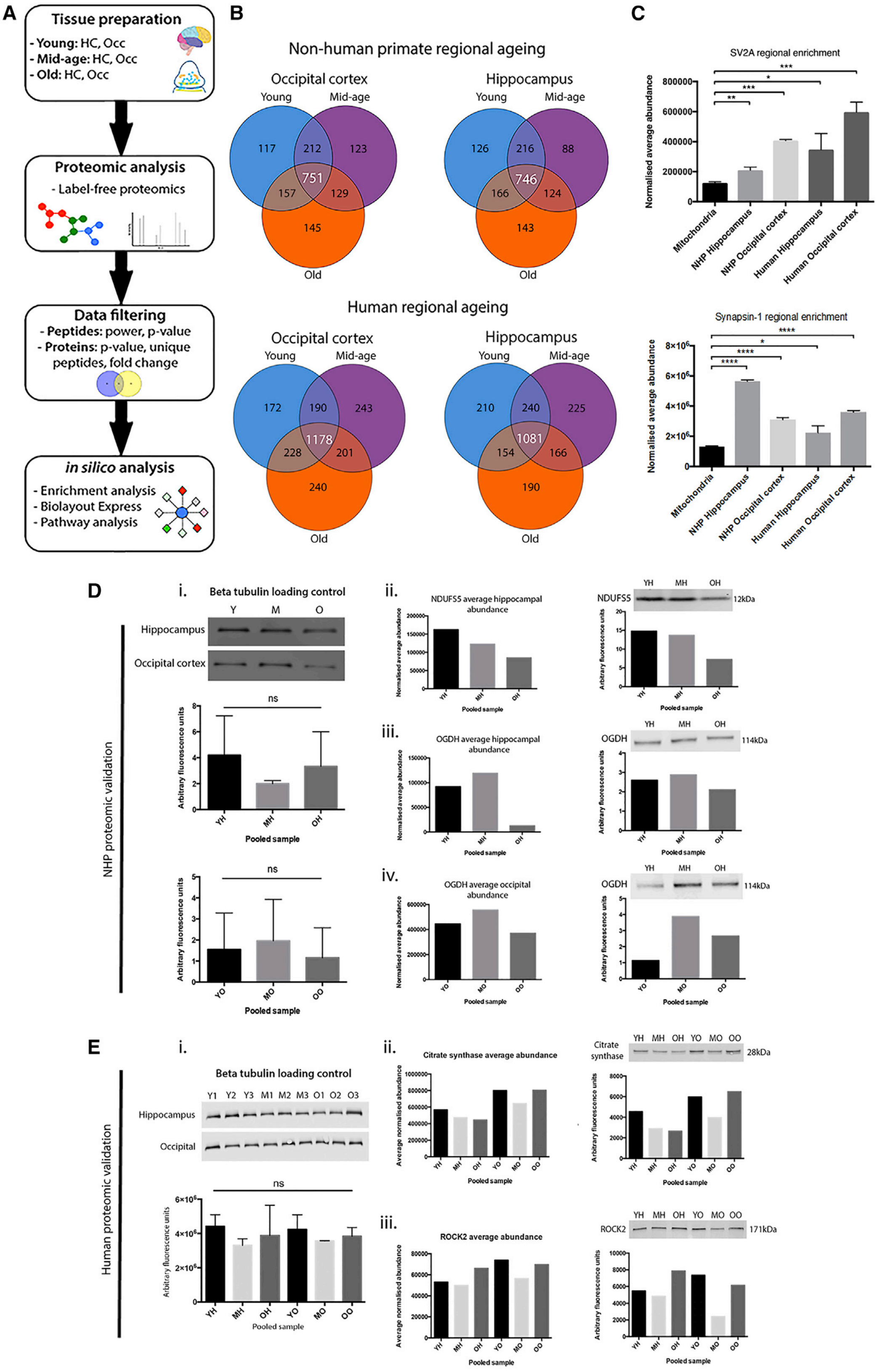

(legend on next page)

the notion that local biochemical alterations likely dictate selective synaptic vulnerability. Temporal proteomic profiling of anatomically distinct age-resistant (occipital cortex) and age-vulnerable (hippocampus) brain regions from the healthy NHP revealed discrete and dynamic alterations in the synaptic proteome, which appear unequivocally conserved in human patients. Spatiotemporal examination of the age-resistant and age-vulnerable synapses using sophisticated *in silico* analyses revealed numerous candidates associated with the age-dependent vulnerability of both the NHP and the human patient hippocampal synaptic milieu. We demonstrate that several of these candidates are constituents of the transforming growth factor β1 (TGF-β1) signaling cascade and that selective activation of TGF-β1 signaling likely mediates the age-dependent vulnerability of both the NHP and the human patient hippocampal synapse.

RESULTS

Proteomic Characterization of Anatomically Defined NHP and Human Patient Synapses

Although it has been well documented that anatomically discrete neuronal populations exhibit divergent levels of vulnerability to age-related alterations during aging, the molecular correlates governing such processes remain to be elucidated. Studies documenting neuronal alterations in primates demonstrate that the occipital cortex appears to be the least affected brain region during aging, with preservation of total neuronal numbers in NHPs (Hof et al., 2000) and volumetric preservation in aged human patients (Raz et al., 2004). Conversely, perturbations in the hippocampal architecture are often associated with advancing age due to the manifestation of Alzheimer's disease in this region (Peters, 2006). Thus, there appears to be a divergent spectrum of synaptic vulnerability upon which the occipital cortex opposes the hippocampus. To determine age-dependent regional molecular alterations occurring in synaptic compartments of the healthy NHP and human patient brain, we initially purified and characterized isolated synaptic preparations (synaptosomes) from differentially vulnerable brain regions (occipital cortex and hippocampus) at 3 time points (young adult, mid-age, and old). Quantitative label-free proteomic analyses identified >1,700 proteins in each region across the time course, revealing dynamic variations in synaptic protein expression. More than 740 proteins were altered by greater than 20% in each discrete region (Figure 1B), demonstrating significant age-dependent biochemical adaptations in both the NHP and the human patient brain.

The purity of regional synaptic preparations was verified with quantitative enrichment analyses using the raw proteomic data (Figures 1C and S1). The normalized average abundance of the well-established synaptic markers synaptic vesicle glycoprotein 2A (SV2A) and synapsin-1 was calculated for each region at the young time point and compared to isolated cortical mitochondria of the same age. To ensure parity between the synaptic samples and the isolated mitochondria, all preparations were loaded onto the mass spectrometer during the same experiment. SV2A and synapsin-1 levels indicated significant enrichment in all respective NHP and human samples versus isolated cortical mitochondria, suggesting purification of synaptic compartments (Figure 1C). In addition to demonstrating purification of synaptic fractions, we employed quantitative fluorescent western blotting (QFWB) to determine the veracity of the proteomic data. We observed corresponding protein expression trends in each tissue preparation, as indicated by the proteomics, for multiple proteins (Figures 1D and 1E). Altogether, these results indicate the relative purity of the synaptic preparations and suggest the proteomic data are representative of the molecular alterations occurring in both NHP and human tissues during aging. To our knowledge, this is the first study to report brain-regional proteomic profiling of primate synapses during the time course of aging.

Regional Heterogeneity in the Synaptic Proteome Is Conserved between Aging NHPs and Aging Human Patients

To address the question of regional vulnerability, we initially sought to determine whether synapses from discrete brain

Figure 1. Regional Characterization of the Synaptic Proteome

(A) Schematic illustrating the experimental design for comparison of differentially vulnerable brain regions throughout the aging time course. HC, hippocampus; OCC, occipital cortex.

(B) Venn diagrams demonstrating regional characterization of the synaptic proteome. Venn diagrams display the total number of proteins identified at discrete time points in each regional analysis during the time course of synaptic aging. Proteins were filtered in Progenesis using the following criteria: $p > 0.05$, <1.2 fold change across the time course, and 1 unique peptide to obtain the proteins that demonstrate the largest alterations during aging. Number of proteins significantly up- or downregulated by >1.2 fold change during the aging time course is indicated at the middle intersection. These filtered proteins were used for all analyses.

(C) Purity of regional synaptic preparations. Purity of regional synaptic isolates was verified with quantitative enrichment analyses using the raw regional proteomic data and isolated cortical mitochondria. Comparative expression of the synaptic markers SV2A and synaptotagmin indicate synaptic enrichment of all regional preparations.

(D and E) Validation of regional temporal proteomic data with quantitative fluorescent western blotting in NHP (D) and human patient (E) samples. (i) Actin loading control for pooled hippocampal and occipital NHP synaptosomes. Samples were pooled according to age group. Bar charts demonstrate there is no significant difference in total protein between ages or regions. (ii–iv) Left bar chart displays the proteomic average normalized expression values of proteins in regional synapses during aging. Right bar chart demonstrates sample protein expression quantified by fluorescent western blots. Proteomic and sample expression of all proteins (hippocampal NDUFS5 [ii], hippocampal OGDH [iii], and occipital cortex OGDH [iv]) follow the same trend, thereby providing validation of the proteomic data.

(E) (i) βIII-tubulin loading control demonstrating equivalent expression between individual patients and between regions. Graph displays no significant difference in βIII-tubulin expression between pooled samples. (ii and iii) Upper bar charts display the proteomic average normalized expression values of the proteins citrate synthase (ii) and ROCK2 (iii) in regional synapses during aging. Lower bar charts demonstrate sample protein expression quantified by the fluorescent western blots shown. Proteomic and sample expression of both citrate synthase and ROCK2 follow the same trend, thereby confirming the proteomic data.

YH, young hippocampus; MH, mid-age hippocampus; OH, old hippocampus; YO, young occipital cortex; MO, mid-age occipital cortex; OO, old age occipital cortex. Statistical analyses used unpaired two-tailed Student's t test (**$p < 0.01$; ***$p < 0.001$; ****$p < 0.0001$).

See Figure S1 for further details.

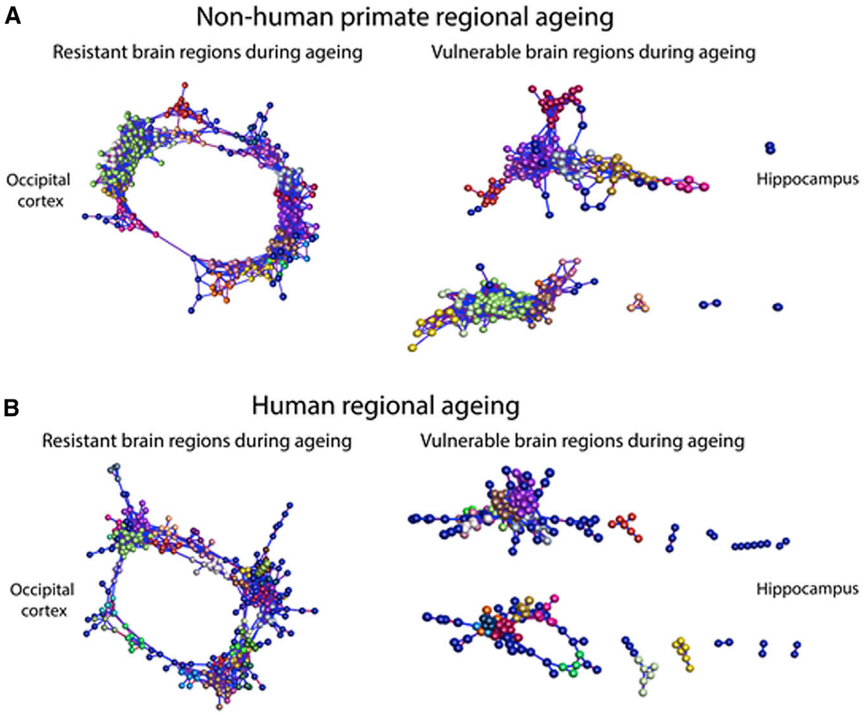

Figure 2. Distinct Clustering Patterns Are Conserved in Human Patient Synapses

(A and B) Regional protein-protein correlation networks displaying proteins significantly altered through the time course of aging in NHPs (A) and human patients (B). Nodes signify individual proteins, and edges reflect the strength of correlation of expression. Colors represent clusters of proteins that are grouped together based on their expression profiles. Correlation networks appear to be conserved in synaptosomes from human patients during normal healthy aging. Graphs are clustered using Pearson r = 0.95.

See Figures S1 and S2 for further details.

regions aged in a similar manner in both NHPs and human patients. Using the objective network visualization software BioLayout Express^{3D} (Theocharidis et al., 2009), we generated brain regional Pearson correlation graphs, in addition to principal-component analyses (PCAs) and heatmaps, demonstrating relative age-dependent similarities (Figure S1). Examination of the Pearson correlation networks and PCAs confirmed distinct region-dependent clustering profiles. Resistant NHP synaptic populations (occipital cortex) exhibited single networks, suggesting congruence among young, mid-age, and old samples (Figure S1); however, the aged synaptic population appears to demonstrate reduced equivalence with the mid-age synapses and enhanced similitude with the young samples (Figures S1 and S2). Similarly, the human patient occipital cortex Pearson correlation graph and PCA biplot demonstrate that mid-age synaptic isolates exhibit reduced correspondence with the young and old time points (Figures S1 and S2). The salient similarities in protein expression between the young and the old occipital cortex synaptic populations may confer resistance to insult during advancing age. Conversely, vulnerable synaptic populations (hippocampus) display fragmentation into 2 smaller networks in both the NHP and the human patient, indicating age-dependent heterogeneity (Figures S1 and S2). Although there appears to be a small degree of variability between the young and the mid-age samples in the NHP (Figure S2), it is evident that the aged hippocampal synaptic population possesses discrete protein expression profiles (Figures S1 and S2), which appear to be conserved in the corresponding human patient samples and may be relevant to the vulnerability status of hippocampal synapses at this particular age (Figure S2). Thus, there are indications that isolated synaptic populations age in a region-dependent manner and these unique alterations may reflect the potential vulnerability of synapses, independent of primate species.

Regional Profiling of Aging Primate Synapses Reveals Protein Expression Trends Correlating with Synaptic Vulnerability

In conjunction with the Pearson correlation graphs and PCAs, to dissect region-specific, age-dependent synaptic alterations, we generated network graphs of the regional time course proteomic data, again using BioLayout Express^{3D} (Theocharidis et al., 2009). The software applies unbiased Markov clustering algorithms to the input data and groups proteins displaying similar expression trends. This allows visualization of spatiotemporal profiles promoting the identification of physiological cascades altered within the dataset. Graphs were constructed using regional differentially expressed proteins (altered >20%) through the time course of aging (Figure 1B), providing 20–30 clusters per region. In agreement with the Pearson correlation graphs and PCAs, network clustering of the proteomic data displayed similar trends with regards to fragmentation of the graphs representing the vulnerable brain regions (hippocampus), whereas synaptic isolates considered resistant during aging appear as one large network (occipital cortex) (Figure 2A). Proteomic data characterizing aging synaptic isolates from human post-mortem samples at equivalent ages to the NHP (young, mid-age, and old) display similar network clustering profiles (Figure 2B). Significant demarcation remains between the occipital cortex and the hippocampus, suggesting resistant and vulnerable brain regions are aging in unique manners in both NHPs and human patients (Figures 2A and 2B). Furthermore, the results suggest that the regional NHP data may be an accurate reflection of human synaptic aging, with clear conservation of protein expression alterations during the time course.

By using two contrasting brain regions demonstrating disparate profiles of aging in both NHPs and human patients, we aimed to identify conserved regulators of synaptic vulnerability with comparison of analogous protein expression profiles. Clusters displaying particular expression trends of interest were selected from both the NHP and the human patient occipital cortex and

hippocampus synaptic time course data using BioLayout Express^{3D} (Figure 3A, 3B, 3E, and 3F). Those exhibiting steady up- or downregulation protein expression profiles during the time course were considered potential biomarkers of normal healthy aging due to the predictable age-dependent tractability of those candidates (Figures 3A and 3E) (for cluster enrichment analyses, see Figure S3). Conversely, proteins displaying late-stage increases or decreases in expression were regarded as potential correlates of regional vulnerability, because the abrupt expression changes observed in the old synaptic populations likely reflected acute alterations disrupting homeostasis at the synapse (Figures 3B and 3F) (for cluster enrichment analyses, see Figure S3). To identify candidates that may be regulating regional synaptic vulnerability during aging in both the NHP and the human patient, proteins from occipital and hippocampal clusters with analogous expression profiles were subject to a subtractive process. Proteins demonstrating equivalent spatiotemporal profiles in both occipital cortex and hippocampal synapses were not considered likely regulators of differential regional synaptic vulnerability and filtered from the data before further analysis (Figures 3C, 3D, 3G, and 3H). Upon subtraction of proteins exhibiting analogous expression profiles, there remained 241 differentially expressed proteins in the NHP analysis and 386 differentially expressed proteins in the human analysis, all of which we tracked through regional aging (Figures 4A and 4B).

The TGF-β1 Signaling Pathway Is a Conserved Cascade Associated with Hippocampal Synaptic Vulnerability in Both Aged NHPs and Human Patients

Although we had characterized spatiotemporal alterations occurring in populations of differentially vulnerable synapses on a global scale, it remained unclear whether the regional divergence in expression of individual candidate proteins may be capable of actively regulating synaptic vulnerability. We reasoned that candidates likely modulating alterations in the stability of the synapse, particularly at old age, would exhibit unequivocal regional temporal profiles. Proteins with corresponding regional expression at the young and mid-age time points followed by a significant demarcation in expression at old age were selected as potential regulators of synaptic vulnerability—these particular expression profiles correlate with previous reports of significant alterations in synapse electrophysiological properties and morphometry in the aged rhesus monkey hippocampus (Morrison and Baxter, 2012; Peters et al., 2008). With characterization of the 241 filtered proteins from the NHP analysis (Figure 4A) and 386 proteins from the human time course (Figure 4B), we identified 17 candidates (NHP candidates: RCN2, CAPZA2, CYC1, OGDH, ROCK2, and CDH2; human candidates: CDC42EP4, STMN3, IGKC, CD99, SLC7A5, APOA1, HCN2, PPT1, FKBP4, HEBP1, and PDAP1) that displayed the archetypal regional spatiotemporal expression profile (Figures 4A and 4B). Thus, these data represent a physiologically relevant paradigm demonstrating that alterations in the expression of these candidates in the NHP and/or human hippocampus may correlate with selective synaptic vulnerability during aging.

Despite presenting several candidates that may have the propensity to alter synaptic morphometry in both NHPs and human patients, it is unlikely that the expression of single proteins solely regulate regional synapse vulnerability during mammalian brain aging. Instead, it is probable that multiple cellular and molecular pathways up- and downstream of the identified candidates converge to regulate age-dependent alterations in synaptic structure and function. By employing Ingenuity Pathway Analysis (IPA) software, we sought to identify common upstream master regulators that may be modulating the expression of the NHP or human patient candidates simultaneously. Despite presenting distinct candidates that may be associated with NHP (RCN2, CAPZA2, CYC1, OGDH, ROCK2, and CDH2) (Figure 4A) or human patient (CDC42EP4, STMN3, IGKC, CD99, SLC7A5, APOA1, HCN2, PPT1, FKBP4, HEBP1, and PDAP1) (Figure 4B) age-dependent hippocampal synaptic vulnerability, upstream analyses highlighted that the identified candidates from both the NHP and the human patient datasets appeared to sit downstream of the microglial cytokine TGF-β1 in the hierarchical cellular signaling cascade (Figures 4C and 4D). With further examination, we established that this particular pathway demonstrated conserved activation in both aged NHP and aged human patient hippocampal synapses, promoting concomitant perturbed expression of our identified candidate proteins (Figures 4C and 4D). Both NHP and human patient occipital cortex synapses of the equivalent age exhibited differential regulation of this pathway, with TGF-β1 showing significant inhibition (Figures 4C and 4D). Thus, there are suggestions that the activation status of TGF-β1 signaling in discrete populations of synapses may be contributing to differential synaptic vulnerability in both NHPs and human patients. TGF-β1 is not constitutively expressed in neurons but instead displays robust levels of expression in microglia (Figure S4), suggesting that regional synaptic vulnerability may be mediated by microglial signaling. Altogether, the data demonstrate that the inherent vulnerability of aged hippocampal synapses may be mediated by perturbations in the microglial TGF-β1 signaling cascade and that this is likely conserved among various primate species.

DISCUSSION

The molecular mechanisms governing the age-dependent decline of selected synaptic populations remain elusive, and studies are just beginning to focus on how protein pathways may synergistically or hierarchically drive this phenomenon. The current investigation is the first of its kind and has enabled the generation of a unique synaptic atlas documenting the age- and region-dependent alterations occurring in the human patient and NHP brain. This comprehensive proteomic profiling has provided a unique insight into how differential age-dependent synaptic vulnerability may be modulated, which may have implications for the field of neurodegenerative diseases. The thorough molecular mapping of both primate species has provided an accessible and dynamic temporal synaptic map to which we may compare disease processes, allowing elucidation of biochemical alterations that may be regarded as pathogenic.

The TGF-β1 Signaling Cascade: A Synaptic-Microglial Intersection?

The identification of fundamental biochemical pathways orchestrating selective synaptic vulnerability is imperative for the

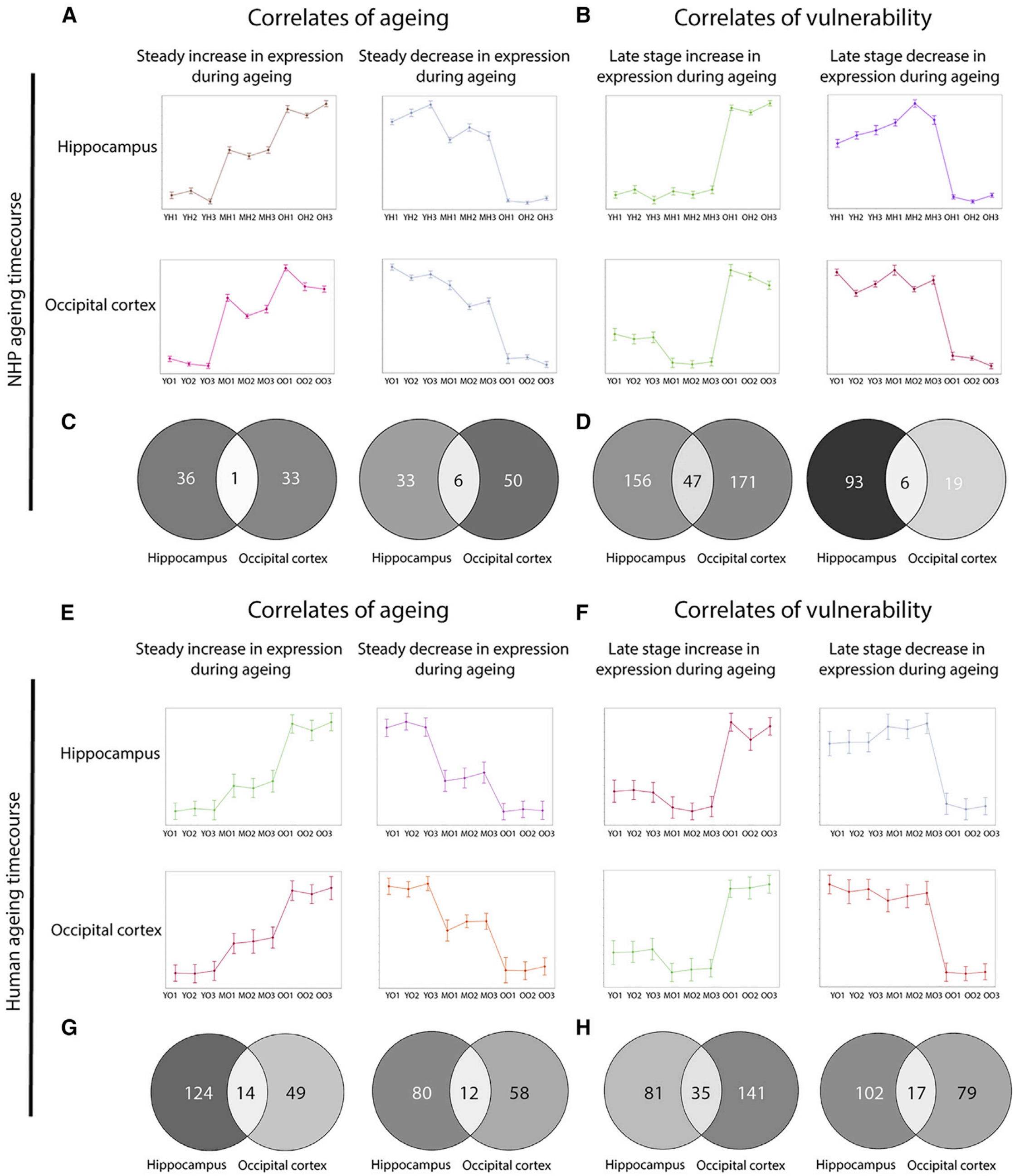

Figure 3. Temporal Profiling of Discrete Synaptic Populations Reveals Biomarkers of Aging and Vulnerability

(A and E) Biomarkers of aging: example temporal expression profiles of proteins demonstrating steady up- or downregulation during aging in the NHP (A) or human patient (E).

(B and F) Biomarkers of vulnerability: example clusters displaying late-stage increases or decreases in temporal protein expression in the NHP (B) or human patient (F). All graphs were generated in BioLayout Express^{3D} (r = 0.95) and display the mean protein expression across the time course in occipital and hippocampal synaptic isolates in both NHPs and human patients.

(C, D, G, and H) Venn diagrams indicating subtraction of candidates. Proteins demonstrating equivalent spatiotemporal profiles in both occipital cortex and hippocampal synapses (shown at the intersection) were not considered regulators of differential regional synaptic vulnerability and were subtracted from further analysis in both the NHP time course (C and D) and the human patient data (G and H).

YH, young hippocampus; MH, mid-age hippocampus; OH, old hippocampus; YO, young occipital cortex; MO, mid-age occipital cortex; OO, old age occipital cortex. Numbers refer to sample technical replicates.

See Figure S3 for further details.

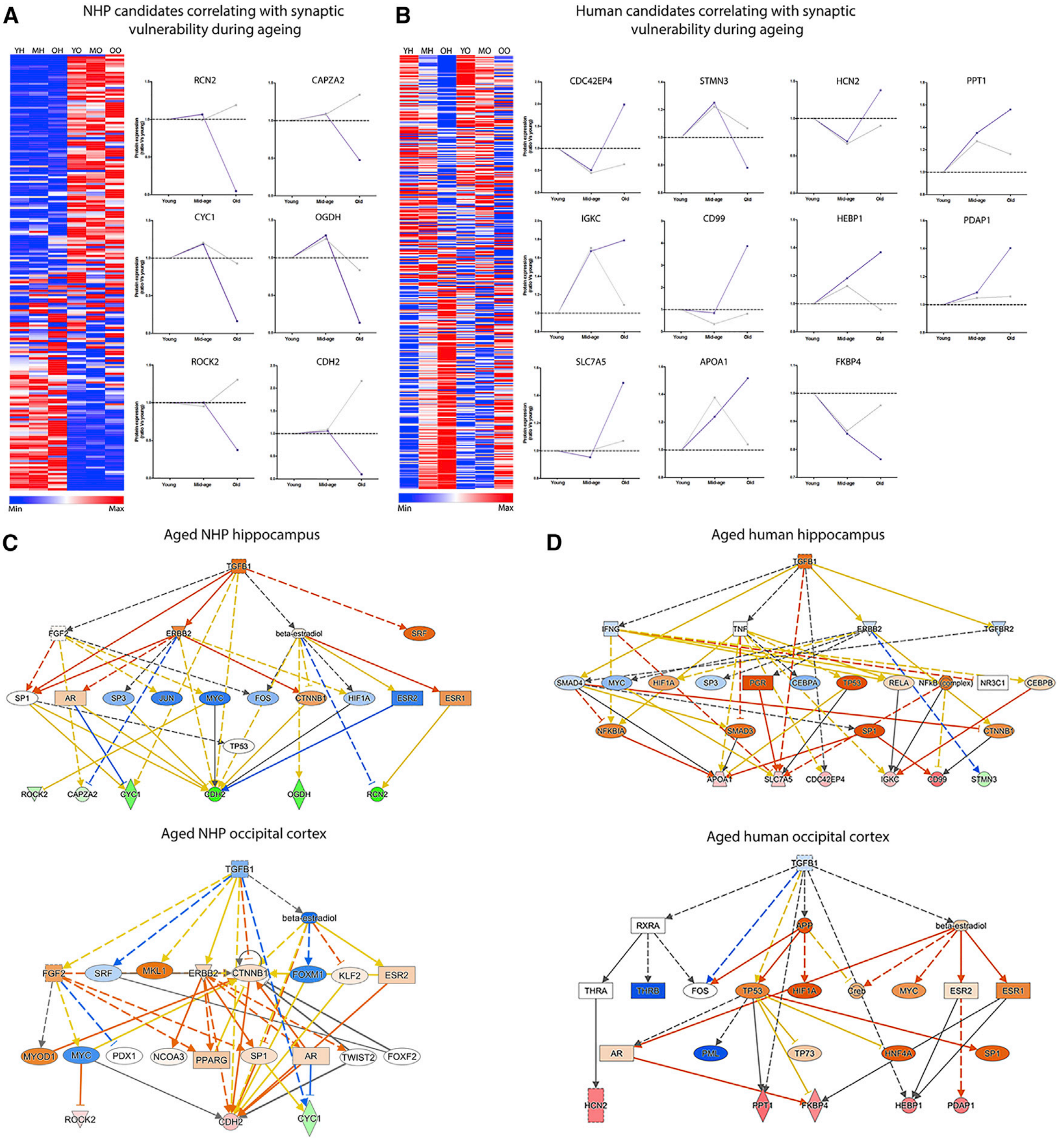

Figure 4. Identification of Potential Candidates and Upstream Regulators Capable of Modulating Synaptic Vulnerability during Aging in the NHP and the Human Patient

(A and B) Right: heatmaps displaying average normalized abundance values of 241 (A) and 386 (B) differentially expressed candidates associated with synaptic vulnerability in both the NHP (A) and the human patient (B). Left: identification of proteins that may have the propensity to modulate regional synaptic vulnerability. Graphs represent temporal expression profiles of selected candidates that likely modulate hippocampal synaptic stability with advancing age. Note the similar expression profiles through young and mid-age in both resistant and vulnerable synaptic compartments, followed by significant divergence in expression at old age. Heatmaps: blue, low expression; red, high expression. Candidate graphs: purple, hippocampal candidate expression during aging; gray, occipital cortex candidate expression during aging.

(C and D) Ingenuity Pathway Analysis (IPA) software highlights TGF-β1 as a conserved common upstream regulator to identified candidates from both the aging NHP (C) and the aging human patient (D) synaptic vulnerability analyses. The TGF-β1 signaling cascade displays significant activation in vulnerable hippocampal synaptic populations at old age, promoting perturbations in candidate expression. Conversely, TGF-β1 signaling appears inhibited in the age-resistant occipital cortex synapses in both NHPs (C) and humans (D). Positive *Z* scores indicate activation (orange), and negative *Z* scores indicate inhibition (blue); green represents downregulation relative to the expression in young animals.

See Figure S4 for further details.

development of neuroprotective strategies. The current investigation suggests there is scope to detect inherent protective modulators, leading to the amelioration of synaptic dysfunction. Despite the candidates displaying significant diversity in localization and function at the synapse, multiple proteins, identified from both the NHP and the human patient analyses, demonstrated a common upstream regulator: TGF-β1. TGF-β1 is not constitutively expressed in neurons but instead displays robust levels of expression in microglia (Figure S4A) suggesting that regional synaptic vulnerability may be mediated by microglial signaling. Investigations have reported age- and region-dependent microglial diversity at the transcriptome level, with indications that hippocampal microglia exhibit compromised function with advancing age (Grabert et al., 2016). In conjunction, immunohistochemical techniques have revealed that microglial populations in aged NHP models and human patients appear to display a dystrophic morphology (Streit et al., 2004). In conjunction with this senescent phenotype, there is abundant evidence to suggest that microglia become hypersensitive or primed, resulting in prolonged pro-inflammatory activation in response to homeostatic alterations (Fan et al., 2017; Udeochu et al., 2016), which may functionally contribute to age-related neuronal alterations via modifications in cytokine signaling and immuno-surveillance (Damani et al., 2011). Furthermore, investigations into the pathogenic role of microglia have documented chronic overexpression of TGF-β1 in multiple neurodegenerative diseases, including Alzheimer's disease, frontotemporal dementia (FTD), amyotrophic lateral sclerosis (ALS), and Parkinson's disease (PD) (Martinez-Canabal et al., 2013; Wirths et al., 2010), illustrating that this pathway may be contributing to numerous age-related diseases in which the synapse is an early pathological target.

Based on this evidence, it is perhaps unsurprising that our data have described microglial TGF-β1 signaling as a potential modulator of regional synaptic vulnerability during aging. Microglia and synapses are not mutually exclusive entities, and there is a requirement for a dynamic and bidirectional relationship to enable homeostatic control of the cellular milieu; however, this critical intersection appears to be potentiating age-dependent instability in the hippocampal synaptic architecture. Despite the wealth of data presented in this study, the cause-consequence relationship underpinning selective synaptic vulnerability remains unresolved. It is unclear whether (1) disruptions in synaptic proteostasis precede aberrant microglial TGF-β1 signaling or (2) microglia are the primary effectors and activation of TGF-β1 signaling facilitates synaptic dysfunction. However, we may speculate that the latter appears to be the most compelling hypothesis, because restoration of upstream microglial signaling promotes synaptic protection in multiple models of aging (Hong et al., 2016; Shi et al., 2017; Daria et al., 2017).

Cumulatively, our data provide comprehensive insight into the regional heterogeneity of synaptic aging and how proteostatic alterations may dictate selective synaptic vulnerability in the primate. Identification of the microglial cytokine TGF-β1 as a master regulator of hippocampal synapse structure and function is indicative of a highly dynamic cohesive cellular network that requires symbiotic modulation for optimal neuronal function during advancing age. Despite this, the implicit role of the TGF-β1 cascade in regional synaptic vulnerability is not unequivocal. It remains unclear whether activation of TGF-β1 signaling is uniquely maladaptive to selected synaptic populations or whether discrete target tissues demonstrate varied allostatic loads. Furthermore, a significant chasm in scientific knowledge concerning microglial-synaptic interactions during advancing age persists, and this must be addressed before we can fully appreciate the functional pathophysiology of age-related cognitive decline.

STAR★METHODS

Detailed methods are provided in the online version of this paper and include the following:

- KEY RESOURCES TABLE
- CONTACT FOR REAGENT AND RESOURCE SHARING
- EXPERIMENTAL MODEL AND SUBJECT DETAILS
  - Ethics
  - Animals
  - Human patient samples
- METHOD DETAILS
  - Synaptosomal preparations
  - Protein concentration assay
  - Label-free proteomics
  - Biolayout Express^{3D}
  - Ingenuity Pathway Analysis
  - Quantitative fluorescent western blotting
- QUANTIFICATION AND STATISTICAL ANALYSIS
  - Statistical analyses
- DATA AND SOFTWARE AVAILABILITY

SUPPLEMENTAL INFORMATION

Supplemental Information can be found online at https://doi.org/10.1016/j.celrep.2019.03.096.

ACKNOWLEDGMENTS

We thank Chris-Anne Mackenzie from the Edinburgh Brain Bank for coordinating the use of human patient tissue and the British consulate in San Francisco for instigating this international collaborative research project. We thank Samantha Eaton and Rachel Kline from the Wishart laboratory for assistance with human sample handling, blotting, and pathway analysis. L.C.G. is supported by a BBSRC studentship and MRC CiC funding. T.M.W. is funded by the BBSRC Institute Strategic Programme (ISPG/5 12-17 and ISPG/1 18-22) and the Euan MacDonald Centre for Motor Neurone Disease Research. S.G.K. and H.F.U. are funded by NIH (OD-011092 and AG-062220).

AUTHOR CONTRIBUTIONS

L.C.G. and T.M.W. conceived the study and designed all experiments. L.C.G. performed experiments. S.G.K. and H.F.U. contributed NHP tissues and reagents. C.S. contributed human tissues. M.J.N. and D.J.L. performed mass spectrometry. All authors edited and approved the manuscript.

DECLARATION OF INTERESTS

The authors declare no competing interests.

Received: October 20, 2017
Revised: September 3, 2018
Accepted: March 26, 2019
Published: April 23, 2019

REFERENCES

Braak, H., and Braak, E. (1995). Staging of Alzheimer's disease-related neurofibrillary changes. Neurobiol. Aging *16*, 271–278, 278–284.

Burke, S.N., and Barnes, C.A. (2006). Neural plasticity in the ageing brain. Nat. Rev. Neurosci. *7*, 30–40.

Clare, R., King, V.G., Wirenfeldt, M., and Vinters, H.V. (2010). Synapse loss in dementias. J. Neurosci. Res. *88*, 2083–2090.

Clinton, J., Blackman, S.E., Royston, M.C., and Roberts, G.W. (1994). Differential synaptic loss in the cortex in Alzheimer's disease: a study using archival material. Neuroreport *5*, 497–500.

Damani, M.R., Zhao, L., Fontainhas, A.M., Amaral, J., Fariss, R.N., and Wong, W.T. (2011). Age-related alterations in the dynamic behavior of microglia. Aging Cell *10*, 263–276.

Daria, A., Colombo, A., Llovera, G., Hampel, H., Willem, M., Liesz, A., Haass, C., and Tahirovic, S. (2017). Young microglia restore amyloid plaque clearance of aged microglia. EMBO J. *36*, 583–603.

Eaton, S.L., Roche, S.L., Llavero Hurtado, M., Oldknow, K.J., Farquharson, C., Gillingwater, T.H., and Wishart, T.M. (2013). Total protein analysis as a reliable loading control for quantitative fluorescent Western blotting. PLoS ONE *8*, e72457.

Fan, X., Wheatley, E.G., and Villeda, S.A. (2017). Mechanisms of Hippocampal Aging and the Potential for Rejuvenation. Annu. Rev. Neurosci. *40*, 251–272.

Fjell, A.M., Amlien, I.K., Sneve, M.H., Grydeland, H., Tamnes, C.K., Chaplin, T.A., Rosa, M.G., and Walhovd, K.B. (2015). The Roots of Alzheimer's Disease: Are High-Expanding Cortical Areas Preferentially Targeted? Cereb. Cortex *25*, 2556–2565.

Gillingwater, T.H., and Wishart, T.M. (2013). Mechanisms underlying synaptic vulnerability and degeneration in neurodegenerative disease. Neuropathol. Appl. Neurobiol. *39*, 320–334.

Grabert, K., Michoel, T., Karavolos, M.H., Clohisey, S., Baillie, J.K., Stevens, M.P., Freeman, T.C., Summers, K.M., and McColl, B.W. (2016). Microglial brain region-dependent diversity and selective regional sensitivities to aging. Nat. Neurosci. *19*, 504–516.

Hof, P.R., Nimchinsky, E.A., Young, W.G., and Morrison, J.H. (2000). Numbers of meynert and layer IVB cells in area V1: a stereologic analysis in young and aged macaque monkeys. J. Comp. Neurol. *420*, 113–126.

Hof, P.R., Gilissen, E.P., Sherwood, C.C., Duan, H., Lee, P.W.H., Delman, B.N., Naidich, T.P., Gannon, P.J., Perl, D.P., and Erwin, J.M. (2002). Comparative neuropathology of brain aging in primates. Interdiscip. Top. Gerontol. *31*, 130–154.

Hong, S., Beja-Glasser, V.F., Nfonoyim, B.M., Frouin, A., Li, S., Ramakrishnan, S., Merry, K.M., Shi, Q., Rosenthal, A., Barres, B.A., et al. (2016). Complement and microglia mediate early synapse loss in Alzheimer mouse models. Science *352*, 712–716.

Josephs, K.A. (2007). Frontotemporal lobar degeneration. Neurol. Clin. *25*, 683–696, vi.

Loerch, P.M., Lu, T., Dakin, K.A., Vann, J.M., Isaacs, A., Geula, C., Wang, J., Pan, Y., Gabuzda, D.H., Li, C., et al. (2008). Evolution of the aging brain transcriptome and synaptic regulation. PLoS ONE *3*, e3329.

Martinez-Canabal, A., Wheeler, A.L., Sarkis, D., Lerch, J.P., Lu, W.Y., Buckwalter, M.S., Wyss-Coray, T., Josselyn, S.A., and Frankland, P.W. (2013). Chronic over-expression of TGFβ1 alters hippocampal structure and causes learning deficits. Hippocampus *23*, 1198–1211.

Morrison, J.H., and Baxter, M.G. (2012). The ageing cortical synapse: hallmarks and implications for cognitive decline. Nat. Rev. Neurosci. *13*, 240–250.

Papazafiri, P., Podini, P., Meldolesi, J., and Yamaguchi, T. (1995). Ageing affects cytosolic Ca2+ binding proteins and synaptic markers in the retina but not in cerebral cortex neurons of the rat. Neurosci. Lett. *186*, 65–68.

Peters, A. (2002). Structural changes that occur during normal aging of primate cerebral hemispheres. Neurosci. Biobehav. Rev. *26*, 733–741.

Peters, R. (2006). Ageing and the brain. Postgrad. Med. J. *82*, 84–88.

Peters, A., Sethares, C., and Luebke, J.I. (2008). Synapses are lost during aging in the primate prefrontal cortex. Neuroscience *152*, 970–981.

Pikkarainen, M., Kauppinen, T., and Alafuzoff, I. (2009). Hyperphosphorylated tau in the occipital cortex in aged nondemented subjects. J. Neuropathol. Exp. Neurol. *68*, 653–660.

Raz, N., Gunning-Dixon, F., Head, D., Rodrigue, K.M., Williamson, A., and Acker, J.D. (2004). Aging, sexual dimorphism, and hemispheric asymmetry of the cerebral cortex: replicability of regional differences in volume. Neurobiol. Aging *25*, 377–396.

Sato, Y., Yamanaka, H., Toda, T., Shinohara, Y., and Endo, T. (2005). Comparison of hippocampal synaptosome proteins in young-adult and aged rats. Neurosci. Lett. *382*, 22–26.

Savli, H., Szendröi, A., Romics, I., and Nagy, B. (2008). Gene network and canonical pathway analysis in prostate cancer: a microarray study. Exp. Mol. Med. *40*, 176–185.

Shi, Q., Chowdhury, S., Ma, R., Le, K.X., Hong, S., Caldarone, B.J., Stevens, B., and Lemere, C.A. (2017). Complement C3 deficiency protects against neurodegeneration in aged plaque-rich APP/PS1 mice. Sci. Transl. Med. *9*, eaaf6295.

Small, S.A., Chawla, M.K., Buonocore, M., Rapp, P.R., and Barnes, C.A. (2004). Imaging correlates of brain function in monkeys and rats isolates a hippocampal subregion differentially vulnerable to aging. Proc. Natl. Acad. Sci. USA *101*, 7181–7186.

Streit, W.J., Sammons, N.W., Kuhns, A.J., and Sparks, D.L. (2004). Dystrophic microglia in the aging human brain. Glia *45*, 208–212.

Theocharidis, A., van Dongen, S., Enright, A.J., and Freeman, T.C. (2009). Network visualization and analysis of gene expression data using BioLayout Express(3D). Nat. Protoc. *4*, 1535–1550.

Udeochu, J.C., Shea, J.M., and Villeda, S.A. (2016). Microglia communication: Parallels between aging and Alzheimer's disease. Clin. Exp. Neuroimmunol. *7*, 114–125.

VanGuilder, H.D., Yan, H., Farley, J.A., Sonntag, W.E., and Freeman, W.M. (2010). Aging alters the expression of neurotransmission-regulating proteins in the hippocampal synaptoproteome. J. Neurochem. *113*, 1577–1588.

Végh, M.J., Rausell, A., Loos, M., Heldring, C.M., Jurkowski, W., van Nierop, P., Paliukhovich, I., Li, K.W., del Sol, A., Smit, A.B., et al. (2014). Hippocampal extracellular matrix levels and stochasticity in synaptic protein expression increase with age and are associated with age-dependent cognitive decline. Mol. Cell. Proteomics *13*, 2975–2985.

Wirths, O., Breyhan, H., Marcello, A., Cotel, M.C., Brück, W., and Bayer, T.A. (2010). Inflammatory changes are tightly associated with neurodegeneration in the brain and spinal cord of the APP/PS1KI mouse model of Alzheimer's disease. Neurobiol. Aging *31*, 747–757.

Wishart, T.M., Paterson, J.M., Short, D.M., Meredith, S., Robertson, K.A., Sutherland, C., Cousin, M.A., Dutia, M.B., and Gillingwater, T.H. (2007). Differential proteomics analysis of synaptic proteins identifies potential cellular targets and protein mediators of synaptic neuroprotection conferred by the slow Wallerian degeneration (Wlds) gene. Mol. Cell. Proteomics *6*, 1318–1330.

STAR★METHODS

KEY RESOURCES TABLE

REAGENT or RESOURCE	SOURCE	IDENTIFIER
Antibodies		
BetaII-tubulin	Abcam	Cat: ab18207; RRID:AB_444319
NDUFS5	ProteinTech	Cat: 15224-1-AP; RRID:AB_2149021
OGDH	ProteinTech	Cat: 15212-1-AP; RRID:AB_2156759
ROCK2	Abcam	Cat: ab71598; RRID:AB_1566688
Citrate synthase	Amsbio	Cat: TA310356
Goat anti-rabbit IRDye 680	Odyssey	Cat: P/N 925-68071; RRID:AB_2721181
Biological Samples		
Healthy rhesus macaque hippocampal tissue blocks of varying ages: young adult (mean age = 9.5 years), mid-age (mean age = 15.6 years), old age (mean age = 23 years); n = *4 animals per age group*	Oregon National Primate Research Center (ONPRC)	N/A
Healthy rhesus macaque occipital cortex tissue blocks of varying ages: young adult (mean age = 9.5 years), mid-age (mean age = 15.6 years), old age (mean age = 23 years); n = *4 animals per age group*	Oregon National Primate Research Center (ONPRC)	N/A
Healthy human patient hippocampal tissue blocks of varying ages: young (18-25 years), mid-age (40-50 years), old age (70+ years); n = 4 samples per age group	Edinburgh Brain and Tissue Bank	N/A
Healthy human patient occipital cortex tissue blocks of varying ages: young (18-25 years), mid-age (40-50 years), old age (70+ years); n = 4 samples per age group	Edinburgh Brain and Tissue Bank	N/A
Chemicals, Peptides, and Recombinant Proteins		
RIPA lysis and extraction buffer	Thermo Fisher	Cat: 89901
Halt Protease inhibitor cocktail (100X)	Thermo Fisher	Cat: 87786
Critical Commercial Assays		
Micro BCA protein assay kit	Thermo Fisher	Cat: 23235
Deposited Data		
NHP hippocampal synaptic aging time course	This paper	https://doi.org/10.7488/ds/2431
NHP occipital cortex synaptic aging time course	This paper	https://doi.org/10.7488/ds/2431
Human patient hippocampal synaptic aging time course	This paper	https://doi.org/10.7488/ds/2431
Human patient occipital cortex synaptic aging time course	This paper	https://doi.org/10.7488/ds/2431
Software and Algorithms		
Xcalibur	Thermo Fisher	Cat: OPTON-30382
Mascot V2.3.2	Matrix Science	http://www.matrixscience.com
Progenesis	Nonlinear Dynamics	http://www.nonlinear.com
BioLayout Express^{3D} *(now known as Graphia Pro)*	Kajeka	https://kajeka.com
Ingenuity Pathway Analysis	QIAGEN	https://www.qiagenbioinformatics.com/products/ingenuity-pathway-analysis

CONTACT FOR REAGENT AND RESOURCE SHARING

Further information and requests for resources and reagents should be directed to and will be fulfilled by the Lead Contact, Dr. Thomas Wishart (t.m.wishart@ed.ac.uk).

EXPERIMENTAL MODEL AND SUBJECT DETAILS

Ethics

In compliance with the 3Rs, no animals were bred specifically for this project. All tissue samples used in the current study were derived from existing archived brains. The Oregon Health & Sciences University Institutional Animal Care and Use Committee at the Oregon National Primate Research Center (ONPRC) approved all animal experiments and the University of Edinburgh ethics committee approved the use of archived material.

Animals

Archived material from 12 rhesus macaques (*Macaca mulatta*) of differing sexes and ages were utilized for the studies described. Animals were assigned to the young adult (mean age = 9.5 years), mid-age (mean age = 15.6 years) or old age (mean age = 23 years) group, with 4 animals per time point. Tissue was historically banked following euthanasia according to procedures recommended by the 2013 Edition of the American Veterinary Medical Association *Guidelines for the Euthanasia of Animals*. Each animal was sedated with ketamine, administered pentobarbital (30 mg/kg, i.v.), and exsanguinated by severance of the descending aorta. Brains were removed and appropriate regional dissections performed before freezing the samples in liquid nitrogen.

Human patient samples

Human patient samples of differing sexes were obtained from the Edinburgh Brain Bank. All tissues were classified as controls due to the absence of gross pathological hallmarks and neurological disease. Human tissues were assigned to equivalent age groups: young (18-25 years), mid-age (40-50 years) or old age (70+ years), with 4 samples per time point. Use of human tissue for post-mortem studies was reviewed and approved by the Edinburgh Brain Bank ethics committee. The Edinburgh Brain Bank is a Medical Research Council funded facility with research ethics committee approval (11/ES/0022).

METHOD DETAILS

Synaptosomal preparations

Regional brain tissue samples were homogenized in an ice-cold isotonic sucrose solution (0.32 M sucrose, 1 mM EDTA, 5 mM Tris-HCl, pH 7.4). Homogenates were centrifuged in a fixed-angle rotor at 900 g for 10 min and the supernatant (S1) was collected. The pellet (P1) was resuspended in sucrose solution and centrifuged again at 900 g for 10 min. Supernatants were combined and centrifuged in a fixed angle rotor at 20,000 g for 15 min. The remaining pellets (P2) contained the synaptosomes.

Protein concentration assay

Samples were homogenized in label-free or RIPA buffer + 1% protease cocktail inhibitor (Thermo Scientific). After homogenization, samples were centrifuged at 20,000 g for 20 minutes at 4°C. The supernatant containing the solubilised protein was removed and pellets discarded. Protein concentration of samples was determined using a Pierce Micro BCA assay kit according to the manufacturer's instructions.

Label-free proteomics

Regional synaptosomal preparations were pooled by brain region and age group (for more information on proteomic methods and the use of pools on human and/or veterinary clinical samples see (19, 27-28)) and extracted in SDT lysis buffer containing 100 mM Tris-HCl (pH 7.6) and 4% (W/V) sodium dodecyl sulfate (VWR). For efficient protein extraction, lysates were freeze–thawed and homogenized in SDT buffer several times. Protein concentration was then determined using BCA assay.

Aliquots (200 μg) of each individual (biological replicates) and pooled (technical replicates) synaptosomal preparation were processed through FASP (filter-aided sample preparation) involving buffer exchange to 8 M urea and alkylation with 50 mM iodoacetamide prior to a double digestion with trypsin (Roche, sequencing grade), initially for 4 h, then overnight at 30°C. Trypsin-digested peptides were separated using an Ultimate 3000 RSLC (Thermo Scientific) nanoflow LC system with the column oven set to 35°C. Technical replicates (3 × ~1 μg) of each pooled sample were loaded at a constant flow of 5 μL/min onto a trapping cartridge (PepMap100, C18, 5 μm, 100Å 0.3 × 5 mm; (Thermo Scientific, San Jose, CA)) using 2% Acetonitrile, 0.1% formic acid. After trap enrichment, peptides were separated on a peptide CSH, 1.7 μm, 130Å, 75 μm x 250mm C18 column (Waters Corp, Milford, MA) with the following gradient: t = 0 min, 2% B; t = 6, 2% B; t = 20, 8% B; t = 110, 24% B; t = 135, 37% B where solvent A is water with 0.1% formic acid and solvent B is 80% acetonitrile with 0.1% formic acid, with a constant flow of 260 nL/min. The HPLC system was coupled to a linear ion trap Orbitrap hybrid mass spectrometer (LTQ-Orbitrap Velos Pro, Thermo Scientific) via a nanoelectrospray ion source (Thermo Scientific). The spray voltage was set to 2.2 kV, and the temperature of the heated capillary was set to 200°C. Full-scan MS survey spectra (*m/z* 335–1800) in profile mode were acquired in the Orbitrap with a resolution of 60 000 after accumulation of 1 000 000 ions. A lock mass of 445.120 024 was enabled for survey scans to improve mass accuracy. The 15 most intense peptide ions from the preview scan in the Orbitrap were fragmented by collision-induced dissociation (normalized collision energy, 35%; activation *Q*, 0.250; and activation time, 10 ms) in the LTQ after the accumulation of 10 000 ions. Dynamic exclusion parameters were set as follows: repeat count, 1; repeat duration, 30 s; exclusion list size, 500; exclusion duration, 45 s; exclusion mass width, plus/minus

10 ppm (relative to reference mass). Maximal filling times were 10 ms for the full scans and 100 ms for the MS/MS scans. Precursor ion charge state screening was enabled, and all unassigned charge states as well as singly charged species were rejected. Data were acquired using Xcalibur software.

Raw proteomic data were imported into Progenesis for characterization and analysis of relative ion abundance. 2D representations of MS/MS output were created for each sample and these were aligned to determine similar features (average alignment score > 80%). Following alignment, data were filtered by retention time with features detected below 17 minutes and above 140 minutes (NHP regional analysis of synaptic aging)/below 11.81 minutes and above 134.37 minutes (human patient regional analysis of synaptic aging) discarded to correct for elution variability. The runs were grouped according to age and brain region and statistical p values were calculated in Progenesis software. For calculation of statistical power and p values, the per run abundances for each grouped experimental condition were transformed through Arc-Sinh normalization to minimize data variability. Following this, 1-way ANOVAs were conducted on the data assigned to the pre-defined experimental conditions resulting in the generation of reliable p values associated with each identified peptide. Peptides were filtered by the following criteria: power < 0.8, fold change > 1.2, p > 0.05 and the remaining data were exported from Progenesis for identification of individual peptide sequences using the IPI-*Macaca mullata* (NHP regional analysis of synaptic aging) and IPI-*Homo sapiens* (human patient regional analysis of synaptic aging) databases via Mascot Search Engine (V2.3.2). Enzyme specificity was set to that of trypsin, allowing for cleavage N-terminal to proline residues and between aspartic acid and proline residues. Other parameters used were as follows. (i) Variable modifications: methionine oxidation, methionine dioxidation, protein N-acetylation, gln → pyro-glu. (ii) Fixed modifications: cysteine carbamidomethylation. (iii) MS/MS tolerance: FTMS- 10 ppm, ITMS- 0.6 Da. (iv) Minimum peptide length: 6. (v) Maximum missed cleavages: 2. (vi) False discovery rate: 1%. A cutoff score of > 34 was used based on Mascot probability threshold of 0.05 that the observed hit is a random event. As an indication of identification certainty, the false discovery rate for peptide matches above identity threshold was set at 1%. Identified proteins were re-imported into Progenesis for further processing. Proteins were subject to stringent filtering parameters to eliminate those which had < 2 unique peptides, < 1.2 fold change between age groups and p > 0.05 to obtain the proteins which demonstrated the largest significant variation in expression over the aging time course in each brain region.

Biolayout Express^{3D}

Proteomic data was dissected using the complex pattern recognition software, Biolayout Express^{3D} (Theocharidis et al., 2009). The software allows visualization of molecular networks by applying Markov clustering algorithms to raw proteomic data (MCL 2.2). All graphs were clustered using Pearson correlation r = 0.95. Clusters of interest indicating age-dependent alterations included those that demonstrated a steady up- or downregulation or a late stage up- or late stage downregulation during the time course of aging. Proteins from clusters with analogous expression profiles underwent a subtractive process – candidates appearing in both the resistant and vulnerable clusters, altered in the same manner, were unlikely to regulate synaptic vulnerability during aging. These candidates were eliminated as modulators of synaptic vulnerability.

Ingenuity Pathway Analysis

IPA was performed as previously described (Wishart et al., 2007) with the interaction data limited as follows: direct and indirect interactions; experimentally observed data only; 35 molecules per network; 10 networks per dataset. Prediction activation scores (z-score) were calculated in IPA. The z-score is a statistical measure of the match between an expected relationship direction and the observed protein expression. Positive z-scores indicate activation (orange) and negative z-scores indicate inhibition (blue) (Savli et al., 2008).

Quantitative fluorescent western blotting

Quantitative fluorescent western blotting was performed as previously described (Eaton et al., 2013). Samples were diluted to provide desired protein concentration. 10-15 μg protein was loaded per well into Nu-PAGE® Novex® 4%–12% Bis Tris mini-gels (Life Technologies) and transferred to PVDF membranes using an iBlot® and Invitrogen gel transfer stacks. Membranes were incubated in primary antibodies at 4°C and secondary antibodies at room temperature (concentrations according to manufacturer's instructions) before imaging on Li-COR Odyssey infrared scanner. Protein expression was quantified utilizing Odyssey software (Li-COR Biosciences). *Primary antibodies: BetaIII-tubulin (Abcam Cat: ab18207), NDUFS5 (ProteinTech Cat: 15224-1-AP), OGDH (ProteinTech Cat: 15212-1-AP), ROCK2 (Abcam Cat: ab71598), Citrate synthase (Origene Cat: TA310356); secondary anitbodies: Goat anti-rabbit IRDye 680 (Odyssey).*

QUANTIFICATION AND STATISTICAL ANALYSIS

Statistical analyses

For calculation of statistical power and p values from the raw proteomic data, the per run abundances for each grouped experimental condition were transformed through Arc-Sinh normalization to minimize data variability. Following this, one-way ANOVAs were conducted on the data assigned to the pre-defined experimental conditions, resulting in the generation of reliable p values associated

with each identified peptide. Peptides were filtered by the following criteria: power < 0.8 and $p > 0.05$ before identification of individual peptide sequences to ensure data harbored sufficient statistical power. For all other experiments, data were collected in Microsoft Excel and statistical tests were performed in GraphPad Prism 6 software. For all analyses $p < 0.05$ was considered significant. Statistical tests used are detailed in the results or figure legends where appropriate.

DATA AND SOFTWARE AVAILABILITY

All proteomic datasets generated and used by this study are freely available at: https://doi.org/10.7488/ds/2431

Cell Reports, Volume *27*

Supplemental Information

Regional Molecular Mapping of Primate Synapses during Normal Healthy Aging

Laura C. Graham, Michael J. Naldrett, Steven G. Kohama, Colin Smith, Douglas J. Lamont, Barry W. McColl, Thomas H. Gillingwater, Paul Skehel, Henryk F. Urbanski, and Thomas M. Wishart

A. NHP ageing timecourse enrichment analysis

PANTHER GO-Slim Cellular Component	Fold Enrichment	P-value
Synapse	5.72	4.05E-03
Vesicle coat	4.57	2.3E-04
Mitochondrial inner membrane	3.63	6.73E-09
Presynaptic membrane	3.48	2.21E-02
Heterotrimeric G-protein complex	3.43	1.33E-02
SNARE complex	3.3	9.26E-03
Postsynaptic membrane	3.2	1.06E-02
Cytoplasmic membrane-bound vesicle	3.05	4.16E-08
Endosome	2.91	3.2E-05

B. Human patient ageing timecourse enrichment analysis

PANTHER GO-Slim Cellular Component	Fold enrichment	P-value
Postsynaptic membrane	10.48	3.97E-02
Mitochondrial inner membrane	5.69	4.06E-09
Ribosome	3.81	2.7E-10
Actin cytoskeleton	3.57	1.02E-12
Cytosol	3.4	2.07E-21
Cell junction	2.89	8.1E-03
Microtubule	2.55	5.63E-03
Cytoskeleton	1.96	1.53E-05
Cytoplasm	1.93	3.66E-29

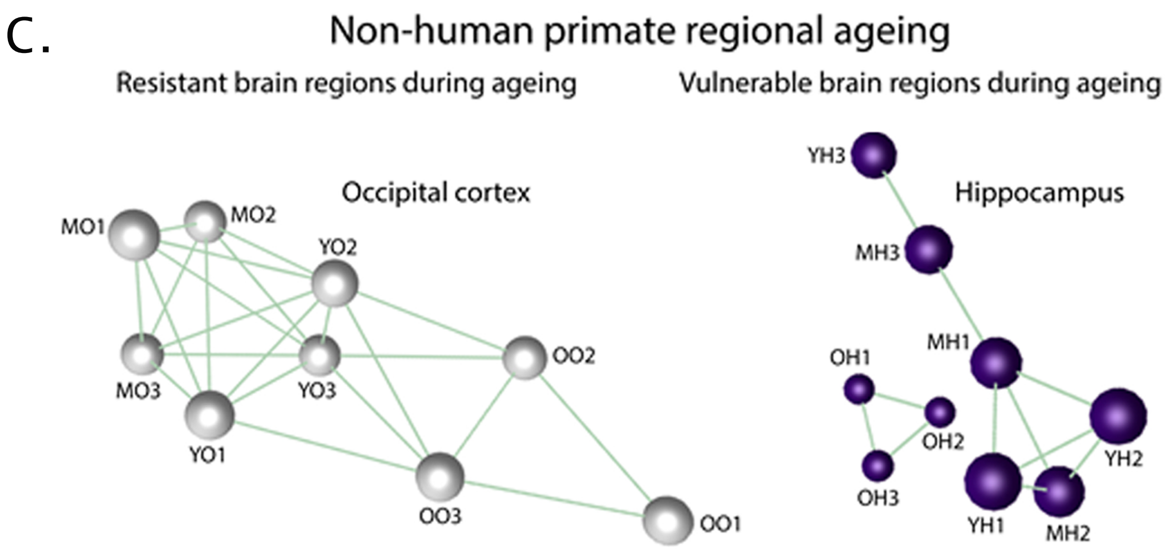

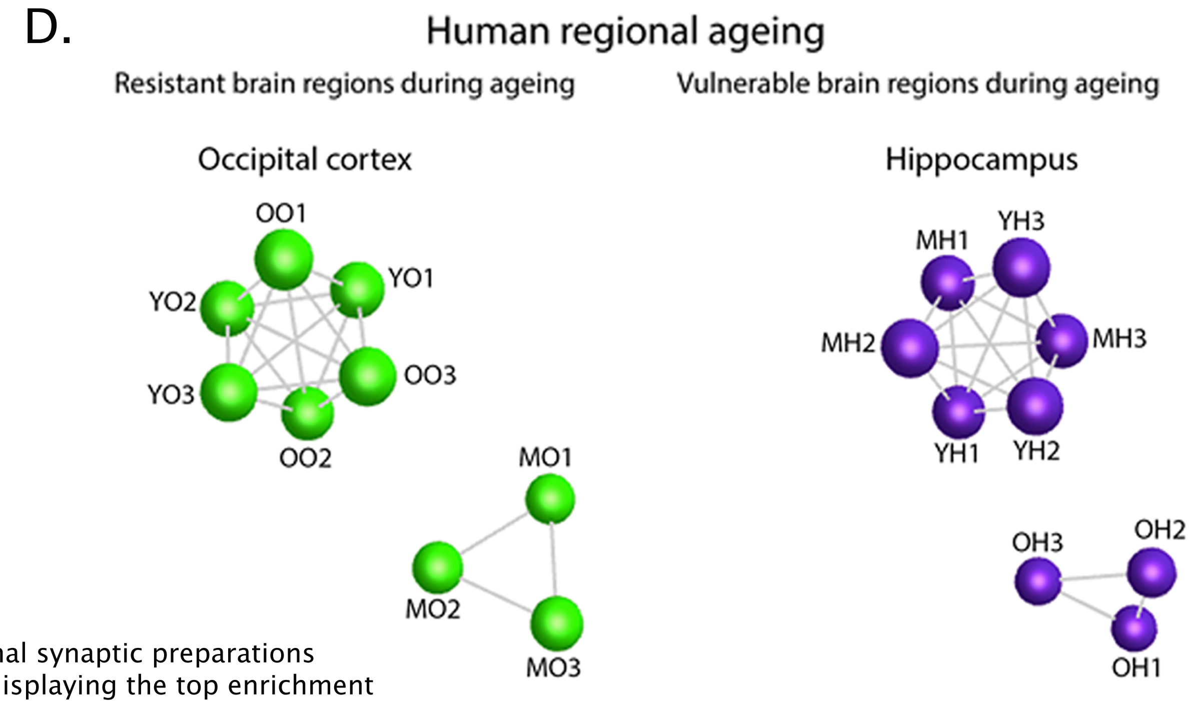

Figure S1 Enrichment analyses demonstrating purity of NHP and human regional synaptic preparations (A&B related to Figure 1): PANTHER Gene ontology (GO) enrichment analyses displaying the top enrichment terms in both the young NHP synaptosomal preparations (A.) and the young human patient synaptic isolates (B.).
Synaptic ageing is regionally heterogeneous. (C&D related to Figure 2): Unbiased sample-sample Pearson correlation analyses generated from BioLayout Express3D software. C. NHP hippocampal and occipital cortex synaptic timecourse Pearson correlation analyses. DA.. NHP hippocampal and occipital cortex synaptic timecourse Pearson correlation analyses. Nodes signify individual samples and edges reflect the strength of correlation of expression. Increased edge length between nodes represents reduced correlative value between sample nodes; reduced edge length between nodes represents increased correlative value between sample nodes; detachment of nodes from primary cluster illustrates significant variability between sample populations. Graphs clustered using Pearson r=0.98.

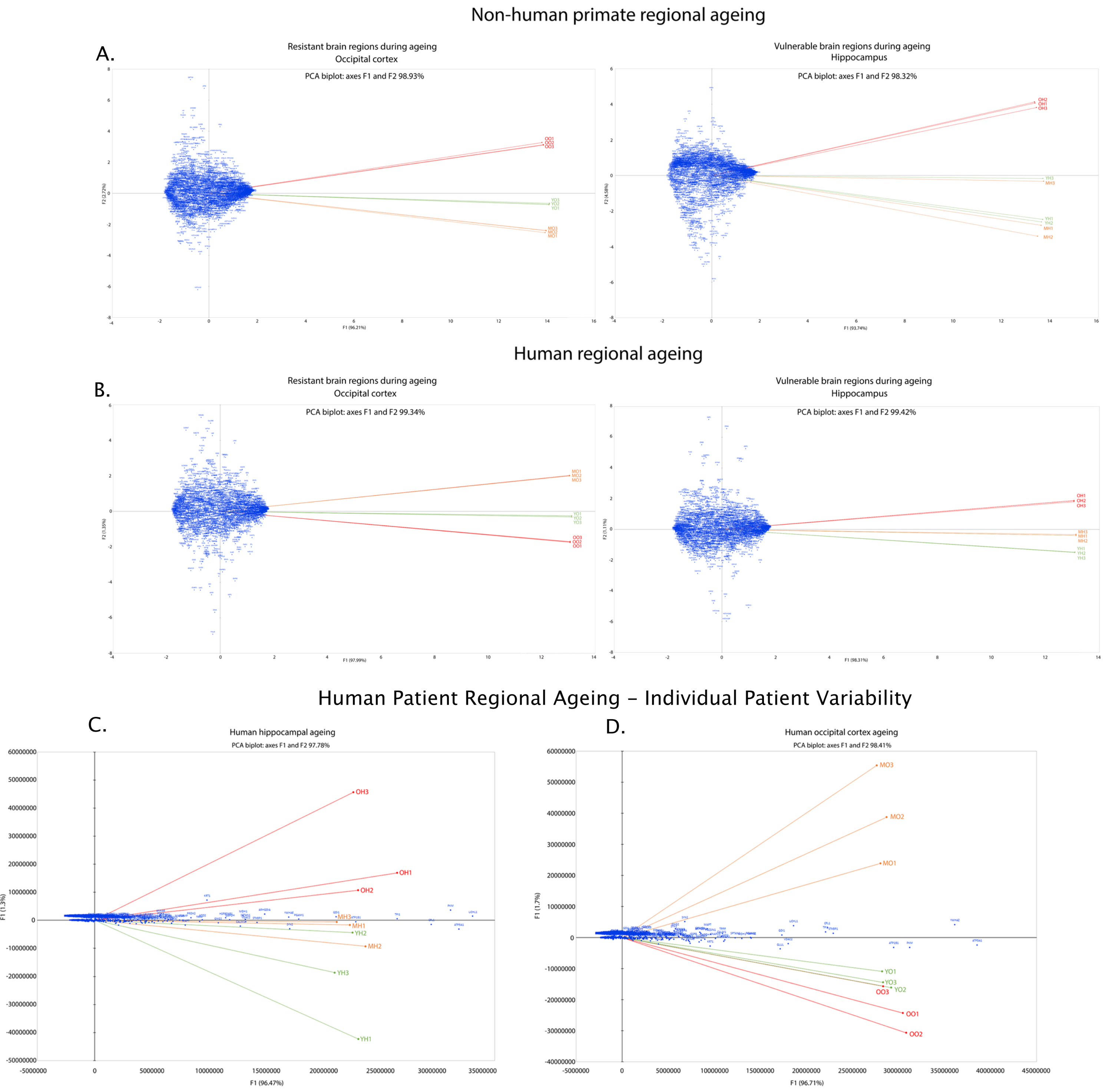

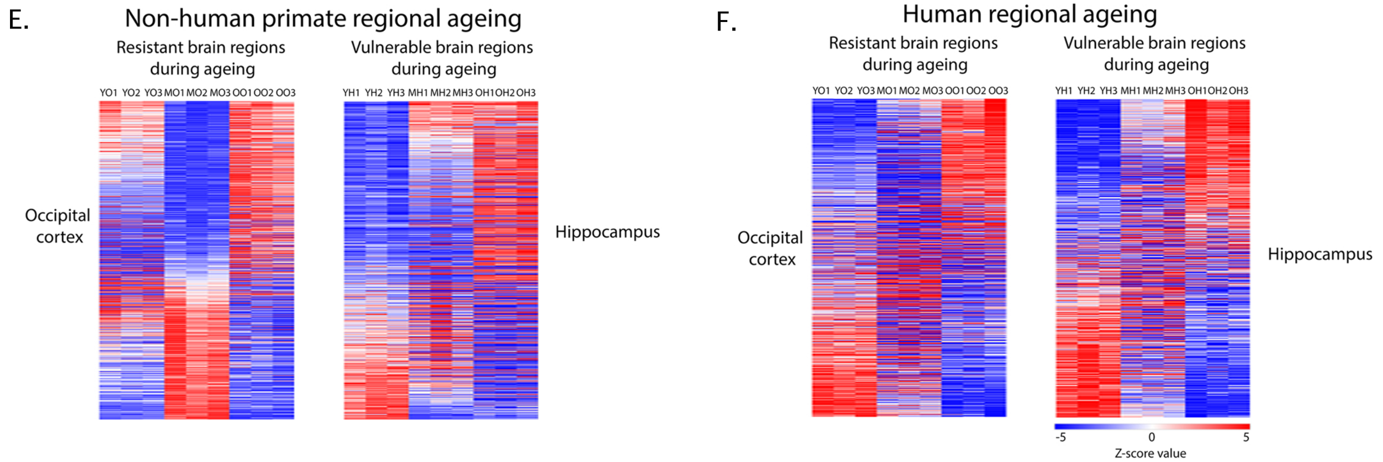

Figure S2: PCA biplots of sample variability (technical replicates – A&B; Example biological replicates – C&D) and heat maps of temporal expression (E&F): PCA biplots conveying global variability in the NHP and human patient brain regional proteomic datasets (related to Figure 2): PCA biplots display identified proteins from the NHP (A) and human patient (B) occipital cortex and hippocampal proteomic datasets. Individual sample technical replicates are indicated by filled circles and grouped by colour. Patient sample technical replicates are indicated by filled circles and grouped by colour: green = young patients; orange = mid-age patients; red = aged patients. Sample variability is dictated by the location of the representative markers in space: proximate variables demonstrate a significant positive correlation (r value is close to or equal to 1); variables opposing the centre line display a significant negative correlation (r value is close to or equal to -1). YO = young occipital cortex; MO = mid-age occipital cortex; OO = old occipital cortex; YH = young hippocampus; MH = mid-age hippocampus; OH = old hippocampus; numbers refer to technical replicates. Blue = active observations in the PCA space (proteins identified by gene name); green, orange and red = active variables observed in the PCA space (green = young time point; orange = mid-age time point; red = aged time point). All PCA biplots generated using Pearson correlation.
C&D (related to Figure 2): PCA biplots displaying inter-patient variability in the human ageing regional proteomic datasets. PCA biplots display 630 identified proteins from the human patient hippocampal (C.) and occipital cortex (D.) proteomic datasets. See above for colour code, abbreviations and generation. Numbers refer to biological replicates. E&F (related to Figure 2): Heat maps illustrating temporal protein expression in differentially vulnerable populations of synapses derived from the NHP and human patient brain. E. Heat maps displaying temporal protein expression in NHP occipital cortex (left panel) and hippocampal (right panel) synaptic populations across ageing.
F. Temporal protein expression in human patient occipital cortex (left panel) and hippocampal (right panel) synapses during advancing age. Heat maps display Z-score transformed normalized abundance values derived from the raw proteomic data. Blue = minimum Z-score value (-5); red = maximum Z-score value (5). YO = young occipital cortex; MO = mid-age occipital cortex; OO = old occipital cortex; YH = young hippocampus; MH = mid-age hippocampus; OH = old hippocampus; numbers refer to technical replicates.

A. Correlates of ageing

B. Correlates of vulnerability

NHP Hippocampus

A. Steady increase during ageing

PANTHER GO Molecular Function	Fold Enrichment	P-value
Phosphatidylcholine-sterol activity	> 100	1.07E-05
Low-density lipoprotein particle binding	33.53	1.64E-04
Amyloid-beta binding	22.01	5.83E-06
Cholesterol binding	20.76	7.56E-06
Calcium-dependent protein binding	19.63	9.67E-06
Oxidoreductase activity	11.18	1.03E-06
Cadherin binding	6.72	3.55E-05
Cell adhesion molecule binding	6.05	3.11E-06
Actin binding	5.04	1.64E-05

A. Steady decrease during ageing

PANTHER GO Molecular Function	Fold Enrichment	P-value
Malate dehydrogenase activity	> 100	1.49E-04
Oxidoreductase activity, acting on NAD(P)H	17.86	7.01E-04
NADH dehydrogenase activity	34.44	1.76E-03
Oxidoreductase activity	3.99	3.76E-03
Ferrochelatase activity	> 100	4.14E-03
Citrate synthase activity	> 100	4.14E-03
Lactoylglutathione lyase activity	> 100	4.14E-03
Methionine-tRNA ligase activity	> 100	6.20E-03
3-oxoacid CoA-transferase activity	> 100	6.20E-03

B. Late stage increase during ageing

PANTHER GO Molecular Function	Fold Enrichment	P-value
Cadherin binding	21.51	5.29E-11
Cell adhesion molecule binding	14.10	2.71E-09
Phospholipase A2 inhibitor activity	> 100	4.22E-05
S100 protein binding	> 100	1.05E-04
Calcium ion binding	5.31	1.30E-04
Calcium-dependent phospholipid binding	31.90	1.44E-04
Phospholipase inhibitor activity	> 100	1.47E-04
Water channel activity	> 100	2.51E-04
Water transmembrane transporter activity	93.04	3.13E-04

B. Late stage decrease during ageing

PANTHER GO Molecular Function	Fold Enrichment	P-value
Dynein intermediate chain binding	41.92	1.23E-03
Protein binding	1.93	3.23E-03
Guanine deaminase activity	> 100	4.32E-03
Prenylcysteine oxidase activity	> 100	6.48E-03
Calcium ion binding	4.11	7.33E-03
Glutaminase activity	> 100	8.63E-03
Ganglioside GT1b binding	> 100	8.63E-03
Sialic acid binding	> 100	8.63E-03
Oxidoreductase activity	> 100	8.63E-03

NHP Occipital cortex

A. Steady increase during ageing

PANTHER GO Molecular Function	Fold Enrichment	P-value
Very-low-density lipoprotein particle binding	> 100	3.28E-05
Phosphatidylcholine-sterol activity	> 100	3.28E-05
Amide binding	15.07	1.49E-04
Antioxidant activity	30.13	1.52E-04
Low-density lipoprotein particle binding	> 100	2.28E-04
Cholesterol transporter activity	63.13	5.45E-04
Sterol transporter activity	57.64	6.45E-04
Sulfur compound binding	18.08	6.50E-04
Oxidoreductase activity	5.49	6.85E-04

A. Steady decrease during ageing

PANTHER GO Molecular Function	Fold Enrichment	P-value
Electron transporter	> 100	1.08E-05
Proton transmembrane transporter activity	20.90	4.22E-05
Transmembrane transporter activity	5.54	2.05E-04
Nucleoside monophosphate kinase activity	73.14	4.09E-04
Inorganic molecule transporter activity	6.00	4.13E-04
Transporter activity	4.82	4.74E-04
Phosphotransferase activity	43.03	1.10E-03
Monocarboxylic acid transporter activity	40.64	1.23E-03
Cation transmembrane transporter activity	8.48	1.23E-03

B. Late stage increase during ageing

PANTHER GO Molecular Function	Fold Enrichment	P-value
Catalytic activity	1.75	9.21E-05
Oxidoreductase activity	11.21	1.05E-04
Transferase activity	> 100	1.08E-04
ATPase regulator activity	34.96	1.26E-04
Cytoskeletal protein binding	3.63	4.71E-04
Ribose phosphate diphosphokinase activity	58.27	7.94E-04
Protein binding	1.69	1.23E-03
Peptidase activity	3.44	1.30E-03
ATPase activator activity	38.85	1.59E-03

B. Late stage decrease during ageing

PANTHER GO Molecular Function	Fold Enrichment	P-value
Calmodulin-dependent protein kinase activity	42.54	6.83E-05
Rac GTPase binding	25.29	2.83E-04
Clathrin binding	20.80	4.87E-04
Rho GTPase binding	9.82	8.24E-04
Cytoskeletal protein binding	3.88	1.08E-03
Spectrin binding	36.70	1.66E-03
ATP-dependent microtubule motor activity	28.36	2.65E-03
Dynein intermediate chain binding	28.36	2.65E-03
Glutamate receptor activity	24.00	3.60E-03

Human Hippocampus

A. Steady increase during ageing

PANTHER GO Molecular Function	Fold Enrichment	P-value
Myosin II binding	54.80	4.88E-05
Aldehyde dehydrogenase (NAD) activity	29.23	2.33E-04
Fatty acid binding	16.70	1.40E-04
Calcium-dependent protein binding	14.14	6.87E-06
Amyloid-beta binding	12.82	6.41E-05
NAD binding	12.38	7.47E-05
Oxidoreductase activity	10.74	6.19E-08
Magnesium ion binding	6.99	2.61E-06
Cadherin binding	4.65	7.67E-05

A. Steady decrease during ageing

PANTHER GO Molecular Function	Fold Enrichment	P-value
NADH dehydrogenase (quinone) activity	49.72	4.46E-14
Oxidoreductase activity	38.12	4.73E-13
Catalytic activity	1.95	8.07E-08
Electron transfer activity	14.17	9.38E-07
Cytochrome-c oxidase activity	33.88	9.67E-06
Heme-copper terminal oxidase activity	33.88	9.67E-06
Proton transmembrane transporter activity	10.02	3.82E-05
Metal cluster binding	13.86	2.48E-04
Structural constituent of presynapse	> 100	2.78E-04

B. Late stage increase during ageing

PANTHER GO Molecular Function	Fold Enrichment	P-value
Phospholipase inhibitor activity	43.12	8.08E-05
S100 protein binding	34.50	1.43E-04
Lipase inhibitor activity	30.44	1.98E-04
Calcium-dependent protein binding	13.91	4.25E-05
Cadherin binding	8.79	7.86E-11
Actin binding	7.27	9.23E-11
Cell adhesion molecule binding	7.16	9.84E-12
Calcium ion binding	4.39	1.83E-07
Cytoskeletal protein binding	4.05	2.71E-08

B. Late stage decrease during ageing

PANTHER GO Molecular Function	Fold Enrichment	P-value
Protein binding	1.54	2.10E-12
Cytoskeletal protein binding	3.90	1.09E-07
ATP-dependent protein binding	65.21	2.91E-05
Structural constituent of ribosome	7.25	6.71E-05
Tubulin binding	4.74	1.46E-04
Glutamate receptor binding	14.49	2.23E-04
Glutaminase activity	> 100	3.21E-04
Chaperone binding	8.61	3.60E-04
Protein self-association	11.79	4.67E-04

Human Occipital cortex

A. Steady increase during ageing

PANTHER GO Molecular Function	Fold Enrichment	P-value
Antioxidant activity	19.04	8.51E-06
Oxidoreductase activity	4.70	2.08E-05
Identical protein binding	2.74	2.77E-04
Coenzyme binding	6.65	3.09E-04
Chaperone binding	12.82	3.10E-04
NAD binding	16.46	9.26E-04
Copper ion binding	15.66	1.06E-03
Cofactor binding	4.37	1.15E-03
Catalytic activity	1.67	1.32E-03

A. Steady decrease during ageing

PANTHER GO Molecular Function	Fold Enrichment	P-value
Oxidoreductase activity	7.33	8.53E-12
NADH dehydrogenase activity	43.53	6.20E-10
Catalytic activity	1.95	2.33E-06
Electron transfer activity	15.52	3.26E-06
Cofactor binding	5.63	1.19E-05
Glutamate:sodium symporter activity	> 100	2.38E-04
Pyruvate dehydrogenase activity	97.42	3.17E-04
Coenzyme binding	6.01	5.38E-04
Glutamate receptor binding	18.27	7.04E-04

B. Late stage increase during ageing

PANTHER GO Molecular Function	Fold Enrichment	P-value
Structural constituent of ribosome	9.33	9.21E-10
RNA binding	2.77	2.80E-09
Identical protein binding	2.59	1.77E-08
Catalytic activity	1.68	2.31E-08
Cadherin binding	5.35	2.59E-07
Structural molecule activity	3.30	4.31E-07
Protein homodimerization activity	3.16	8.74E-07
Oxidoreductase activity	8.19	2.75E-06
Ion binding	1.49	2.81E-05

B. Late stage decrease during ageing

PANTHER GO Molecular Function	Fold Enrichment	P-value
Cytoskeletal protein binding	5.37	9.97E-11
Structural constituent of synapse	57.18	1.54E-06
Protein binding	1.42	4.01E-06
Catalytic activity	1.76	1.41E-05
Actin filament binding	8.43	2.47E-05
Calmodulin binding	8.38	2.55E-05
Proline-rich region binding	34.31	1.33E-04
Clathrin binding	15.51	1.65E-04
Structural molecule activity	3.37	2.44E-04

Figure S3 (related to Figure 3): Enrichment analyses displaying the predominant molecular functions of identified Biolayout Express3D clusters. PANTHER GO Enrichment tables demonstrating the primary molecular functions of identified clusters termed “biomarkers of ageing” (A.) or “biomarkers of vulnerability” (B.) in synaptic isolates derived from both the NHP and human patient hippocampus and occipital cortex. Note the difference in enrichment terms between resistant (occipital cortex) and vulnerable (hippocampus) brain regions within species. Additionally, note the identification of similar enrichment terms in analogous brain regions independent of species.

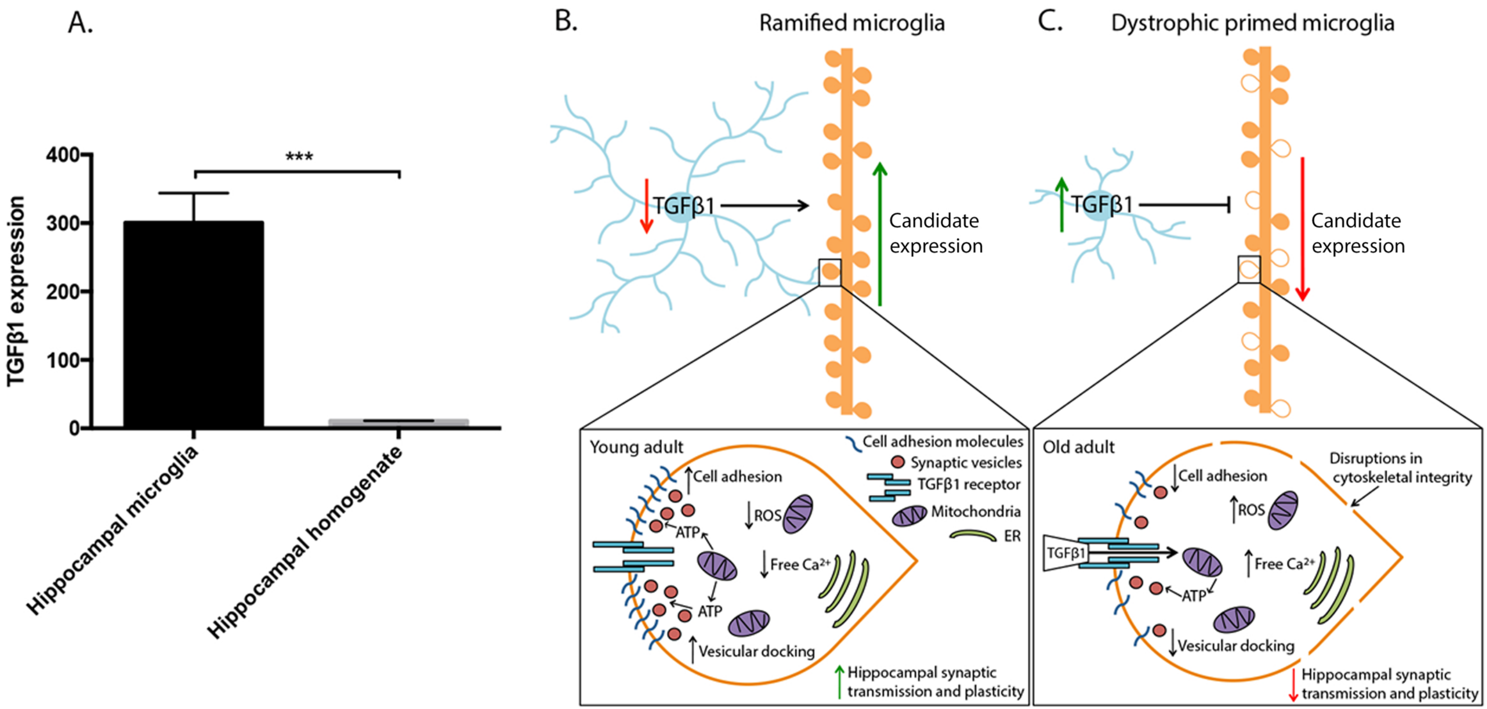

Figure S4 (related to Figure 4): TGFβ1 is expressed exclusively in microglial isolates. A. Bar chart displaying TGFβ1 transcript expression in isolated hippocampal microglia and hippocampal homogenate. The homogenate exhibits relatively little TGFβ1 expression versus the isolated microglia (unpaired two-tailed student's t-tests, $p=0.0009$; data represent mean SEM). B&C. Summary of synaptic-microglial interactions and regulation of the cellular milieu during ageing. Young adult microglia (B.) demonstrate ramified morphology and inhibition of the TGFβ1 signalling cascade, promoting homeostatic regulation of the subcellular machinery within the synaptic compartment. Intracellular processes are illustrated in the black box. Conversely, resident microglia in an aged brain display a dystrophic, primed phenotype (C.). Activation of the TGFβ1 signalling cascade, by binding of TGFβ1 to the TGFβ1 synaptic receptor, promotes injurious events within the synaptic terminal facilitating synapse instability. Orange colour represents dendritic arbour with synaptic terminals; pale blue indicates microglia; white synaptic terminals symbolises dysfunction. See key in black box for intracellular information. YH – young hippocampus; MH – mid-age hippocampus; OH – old hippocampus; YO – young occipital; MO – mid-age occipital; OO – old age occipital.
